# Partial in vivo reprogramming enables injury-free intestinal regeneration via autonomous *Ptgs1* induction

**DOI:** 10.1126/sciadv.adi8454

**Published:** 2023-11-24

**Authors:** Jumee Kim, Somi Kim, Seung-Yeon Lee, Beom-Ki Jo, Ji-Young Oh, Eun-Ji Kwon, Keun-Tae Kim, Anish Ashok Adpaikar, Eun-Jung Kim, Han-Sung Jung, Hwa-Ryeon Kim, Jae-Seok Roe, Chang Pyo Hong, Jong Kyoung Kim, Bon-Kyoung Koo, Hyuk-Jin Cha

**Affiliations:** ^1^ College of Pharmacy, Seoul National University, Seoul, Republic of Korea.; ^2^ Department of Life Sciences, Pohang University of Science and Technology (POSTECH), Pohang 37673, Republic of Korea.; ^3^ Division in Anatomy and Developmental Biology, Department of Oral Biology, Taste Research Center, Oral Science Research Center, BK21 FOUR Project, Yonsei University College of Dentistry, Seoul, South Korea.; ^4^ Department of Biochemistry, Yonsei University, Seoul, Korea.; ^5^ Theragen Bio Co., Ltd, Seongnam 13488, Republic of Korea.; ^6^ Center for Genome Engineering, Institute for Basic Science, 55, Expo-ro, Yuseong-gu, Daejeon 34126, Republic of Korea.; ^7^ Institute of Molecular Biotechnology of the Austrian Academy of Sciences (IMBA), Vienna Biocenter (VBC), Dr. Bohr-Gasse 3, Vienna 1030, Austria.

## Abstract

Tissue regeneration after injury involves the dedifferentiation of somatic cells, a natural adaptive reprogramming that leads to the emergence of injury-responsive cells with fetal-like characteristics. However, there is no direct evidence that adaptive reprogramming involves a shared molecular mechanism with direct cellular reprogramming. Here, we induced dedifferentiation of intestinal epithelial cells using OSKM (Oct4, Sox2, Klf4, and c-Myc) in vivo. The OSKM-induced forced dedifferentiation showed similar molecular features of intestinal regeneration, including a transition from homeostatic cell types to injury-responsive–like cell types. These injury-responsive–like cells, sharing gene signatures of revival stem cells and atrophy-induced villus epithelial cells, actively assisted tissue regeneration following damage. In contrast to normal intestinal regeneration involving *Ptgs2* induction, the OSKM promotes autonomous production of prostaglandin E2 via epithelial *Ptgs1* expression. These results indicate prostaglandin synthesis is a common mechanism for intestinal regeneration but involves a different enzyme when partial reprogramming is applied to the intestinal epithelium.

## Teaser

Partial reprogramming achieves intestinal regeneration through a common molecular mechanism of injury-induced dedifferentiation.

## INTRODUCTION

In the intestinal epithelium, Lgr5+ crypt base columnar cells (CBC cells) play the workhorse stem cell role by constantly producing progenitors and differentiated cells during homeostasis (*1*, *2*). As they are constantly undergoing cell division, Lgr5+ CBC cells are highly sensitive to genotoxic stresses such as ionizing radiation (IR). Upon IR-induced crypt damage, the cycling CBC cells are completely lost, yet the intestinal epithelium is still regenerated and can restore the Lgr5+ CBC cells (*3*). Various reserve stem cells and injury-responsive cells have been proposed, including secretory progenitors (*4*–*6*), label-retaining +4 cells (*3*, *7*), and revival stem cells (revSCs) (*8*). The consensus among these studies is that the intestinal epithelium has various populations of cells with varying degrees of plasticity, which can be recruited rapidly for the optimal healing process.

With the advent of single-cell RNA sequencing (scRNA-seq) technology, injury-responsive cells in the intestinal epithelium have been characterized, leading to the discovery of revSCs (*8*) and atrophy-induced villus epithelial cells (aVECs) (*9*). They appear in different parts of the intestinal units, i.e., crypts and villi, but share molecular characteristics: cellular plasticity, a fetal-like gene expression profile and yes-associated protein (YAP) activation (*8*, *9*). Because of the induction of a fetal gene expression program, the intestinal epithelial injury response is believed to involve dedifferentiation and induction of plasticity through natural adaptive reprogramming in vivo (*10*).

Cellular plasticity and dedifferentiation of somatic cells up to pluripotency can be induced by prolonged expression of the “Yamanaka factors,” Oct4, Sox2, Klf4, and c-Myc (hereafter OSKM) (*11*), through epigenetic reprogramming such as progressive and conserved global erasure of DNA methylation (*12*). OSKM-mediated cellular reprogramming is the gold-standard method for the establishment of induced pluripotent stem cells and has been widely used for various applications (*13*). More recently, several studies demonstrated that forced partial reprogramming with OSKM enables rejuvenation or fetal-like dedifferentiation of cells in the eye, muscle, heart, and liver by inducing youthful DNA methylation patterns and transcriptomes (*14*) and activating muscle stem cells and cardiomyocytes (*15*–*17*). However, no mechanistic insight has been revealed to account for the regenerative effect of partial reprogramming in various tissues.

Here, we hypothesized that the natural dedifferentiation and acquisition of plasticity (i.e., natural adaptive reprogramming) that are induced by intestinal tissue injury share common features of forced partial reprogramming, as they also induce a fetal-like phenotype with improved regeneration capacity. To test this hypothesis, we induced dedifferentiation of intestinal epithelial cells by forced partial reprogramming through the induction of OSKM in vivo. Through extensive scRNA-seq analysis, we found that OSKM drives the induction of dedifferentiation, resulting in injury-responsive–like cells (e.g., revSC- and aVEC-like cells) similar to natural adaptive reprogramming process. These OSKM-induced dedifferentiation facilitated intestinal injury repair. Both “injury-induced” and “OSKM-induced” dedifferentiation showed prostaglandin synthesis as a common underlying mechanism responsible for the regeneration. However, the OSKM-induced dedifferentiation involved the ectopic epithelial expression of prostaglandin-endoperoxide synthase 1 (*Ptgs1*), not via mesenchymal activation of *Ptgs2* as for natural regeneration (*18*). We found that *Ptgs1*-mediated epithelial prostaglandin production is the key event for in vivo partial reprogramming, which directly renders intestinal epithelial cells to adopt fetal characteristics without involving any mesenchymal assistance.

## RESULTS

### Dedifferentiation of intestinal epithelium by partial reprogramming

**Fig. 1.**
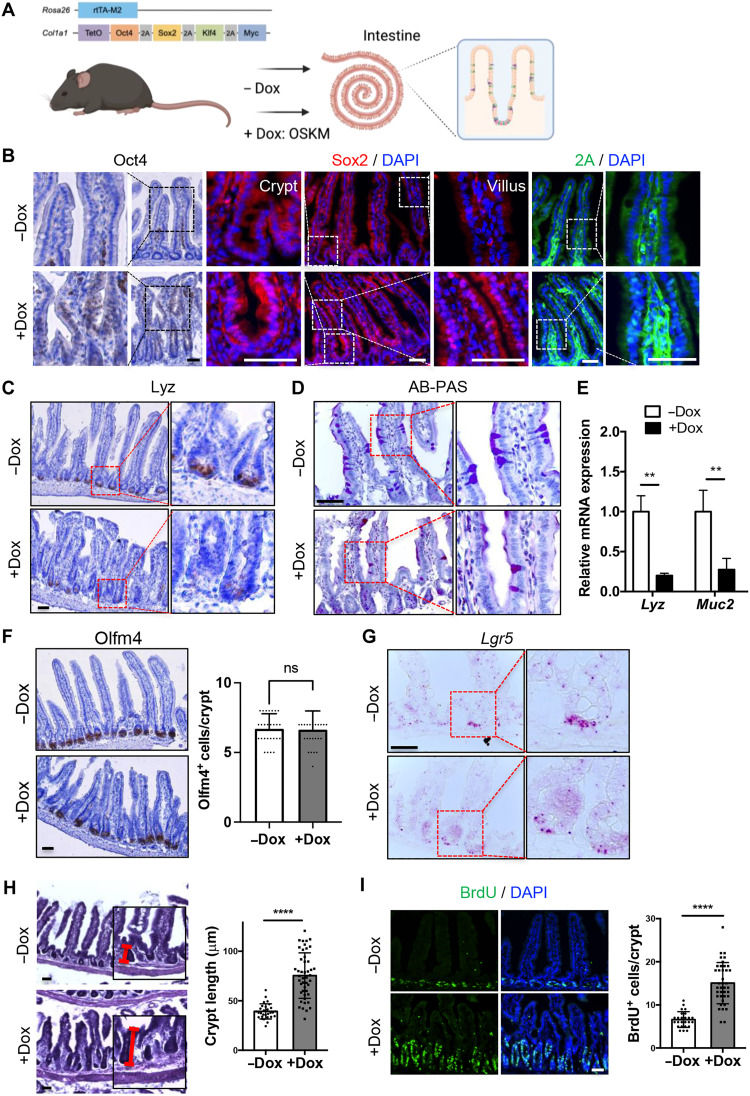
Dedifferentiation of intestinal epithelium by partial reprogramming. (**A**) Experimental scheme on the intestine of Dox inducible OSKM (iOSKM) mouse model. (**B**) Immunohistochemistry (IHC) of Oct4 and immunofluorescence (IF) of Sox2 and 2A peptide in the intestine of iOSKM mice. Samples were analyzed 4 days after Dox administration. Hematoxylin or DAPI for nuclear staining. (**C**) IHC of Lysozyme for PC in the intestine of iOSKM mice. (**D**) AB-PAS staining for GC in the intestine of iOSKM mice. (**E**) Relative mRNA expressions of *Lysozyme* (*Lyz* for PC) and *Mucin 2* (*Muc2* for GC) in intestinal epithelial cells of iOSKM mice. *n* = 6; 3 mice × 2 technical replicates. (**F**) IHC of Olfm4 in the intestine of iOSKM mice (left) and the quantification of Olfm4+ cells per crypt (−Dox, *n* = 30; +Dox, *n* = 28 in total two mice of each condition). (**G**) RNA scope of *Lgr5* in the intestine of iOSKM mice (**H**) H&E histology in the intestine of iOSKM mice. Red line indicates the length of crypt (left). Quantification of crypt length (−Dox, *n* = 29; +Dox, *n* = 46 in total three mice of each condition) (right). (**I**) IF of BrdU in the intestine (left) and quantification of BrdU-positive cells per crypt (−Dox, *n* = 25; +Dox, *n* = 39 in total three mice of each condition) (right). BrdU was administered 3 hours before the euthanasia. Data represent the mean with SD. Student’s *t* test: **P < 0.01 and ****P < 0.0001; ns, not significant. Scale bar, 50 μm.

Unlike chronic OSKM expression, which induces multiple teratomas in mice (*19*), transient OSKM induction (or partial reprogramming) improves aging phenotypes without tumor formation (*20*, *21*). We induced OSKM in the intestine (fig. S1A) by doxycycline (Dox) administration for 4 days with neither tumor formation (fig. S1B) nor weight loss (fig. S1C) in the “reprogrammable mouse” model (inducible OSKM: iOSKM) (*22*) (Fig. 1A). Oct4 and Sox2 proteins, which are rarely expressed in the intestine, were induced throughout the intestinal epithelium, corresponding to clear 2A peptide expression after Dox challenge (Fig. 1B). Upon transient OSKM expression, differentiated cells such as Paneth cells [(PCs; positive for lysozyme staining (Lyz)] and goblet cells [GCs; positive for Alcian blue–periodic acid-Schiff staining (AB-PAS)] appeared diminished (Fig. 1, C and D), which was consistent with repression of marker genes for PCs (i.e., *Lyz*) and GCs (i.e., *Muc2*) (Fig. 1E). The number of CBC cells, marked by olfactomedin-4 (Olfm4) (*23*), was not significantly altered (Fig. 1F), despite a notable reduction of Lgr5, a Wnt-dependent CBC marker (Fig. 1G). The Dox-challenged intestine exhibited a hyperplastic morphology with lengthened crypt region (Fig. 1H) and increased numbers of proliferating cells (Fig. 1I and fig. S1D).

### Cellular and molecular alterations induced by partial reprogramming

**Fig. 2.**
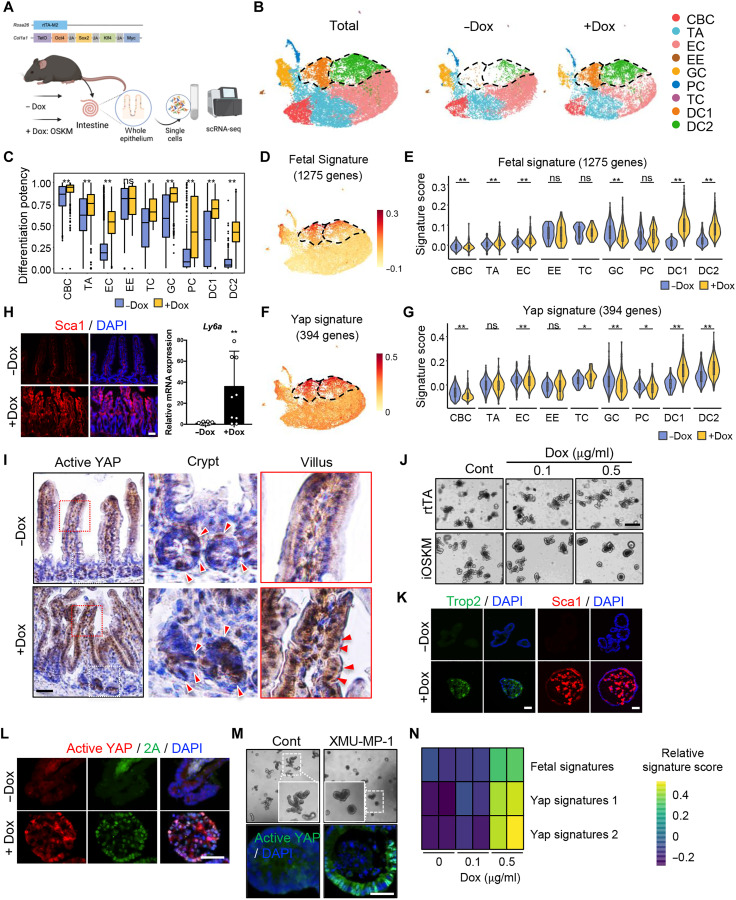
Cellular and molecular alterations induced by partial reprogramming. (**A**) Experimental scheme for scRNA-seq using intestine of iOSKM mouse in Dox condition. (**B**) UMAP plots of scRNA-seq from OSKM-induced mouse intestinal epithelium in −Dox and + Dox conditions (Total, left), −Dox (control, middle), and + Dox (Dox-treated, right). (**C**) Bar plot indicating differentiation potency inferred by CytoTRACE per condition in different cell types. (**D** and **E**) UMAP plot (D) and violin plot (E) showing gene module score of 1275 fetal signature genes. (**F** and **G**) UMAP plot (F) and violin plot (G) showing gene module score of 394 yap signature genes. (**H**) IF of Sca1 in the intestine. DAPI for nuclear staining (left). Relative mRNA expression of *Ly6a* (encoding Sca1) in intestinal epithelial cells of iOSKM mice after Dox treatment (right). (**I**) IHC of Active YAP in the intestine of iOSKM mice 4 days after Dox administration. Red arrowheads indicate active YAP. (**J**) Microscopic images of Dox-treated intestinal organoids from rtTA mouse or iOSKM mouse. (**K**) IF of fetal markers, Trop2 and Sca1, in iOSKM intestinal organoids with Dox treatment for 3 days. DAPI for nuclear staining. (**L**) IF of Active YAP and 2A peptide in iOSKM intestinal organoids. DAPI for nuclear staining. (**M**) Microscopic images (top) and IF of Active YAP (bottom) in XMU-MP-1 (Mst1/2 inhibitor)–treated intestinal organoids. (**N**) Gene enrichment of YAP or fetal signatures in control and iOSKM intestinal organoids. Data represent the mean with SD. Student’s *t* test: *P < 0.05 and **P < 0.01. Scale bar, 50 μm [(H), (I), (K), (L), and (M)], 500 μm (J).

To characterize the cellular and molecular alterations induced by forced partial reprogramming, we performed scRNA-seq on intestinal epithelial cells with or without Dox administration (Fig. 2A). On the basis of our computational pipeline for quality control (QC) and batch correction, we generated a partially reprogrammed intestine atlas of 17,000 QC-positive epithelial cells, with an average of 2473 genes and 12,277 unique molecular identifiers (UMIs) per cell (fig. S1E). We applied uniform manifold approximation and projection (UMAP) for visualization and a graph-based clustering algorithm to identify 18 cell clusters (fig. S1F). On the basis of the expression of canonical marker genes, we identified seven canonical cell types including crypt-base columnar cells (CBC), transit-amplifying cells (TA), enterocytes (EC), enteroendocrine cells (EE), goblet cells (GC), Paneth cells (PC) and tuft cells (TC), as well as two distinct Dox-induced subsets (DC1 and DC2) that were manifest after Dox treatment (Fig. 2B, and fig. S1, G and H). DC1 did not express any canonical intestinal epithelial cell markers, while DC2 partially expressed EC-related marker genes (fig. S1G).

We next examined the molecular alterations induced by forced partial reprogramming for each cell type. By measuring the extent of stemness or dedifferentiation potential, we found that most cell types except EE and TC showed significantly elevated stemness scores upon Dox treatment (Fig. 2C and fig. S1I). In addition, the expression of canonical marker genes for EE, GC, PC, TC (fig. S1J), and CBC (fig. S1K) was decreased upon Dox treatment compared to their corresponding cell types, in congruence with the observations in Fig. 1 (C, D, and G). The induction of a fetal gene signature was most evident in DC1 and DC2 clusters (Fig. 2, D and E, and table S1), along with an enriched Yap signature (Fig. 2, F and G, and table S1). It is noteworthy that the acquisition of fetal phenotypes during injury repair in the intestine also occurs in a Yap-dependent manner (*24*). As predicted, we also observed induction of Sca1 (Fig. 2H) and Trop2 (fig. S1L), encoded by *Ly6a* and *TACSTD2*, typical fetal genes expressed in the intestine after injury (*24*–*26*), along with a pronounced nuclear translocated active Yap (unphosphorylated Yap) signal (*27*) from the crypt and villi upon Dox treatment (Fig. 2I). These results suggest that forced partial reprogramming induces emergence of two distinctive populations with fetal-like characteristics and YAP activation (DC1 and DC2) as well as cellular plasticity of diverse cell types.

Next, to examine the effect of OSKM directly on the intestinal epithelium, we established an intestinal organoid model from the reprogrammable mouse. Transient OSKM expression for 3 days (fig. S2, A and B) was insufficient to induce *Nanog*, a marker for full reprogramming, even with Dox (0.5 μg/ml) (fig. S2C), confirming that reprogramming was only partial and pluripotency was not induced. There was a clear morphological change from budding organoids to cystic spheroids upon Dox treatment in a dose-dependent manner, compared to Dox-treated intestinal organoids from *Rosa26-rtTA* mouse as a control of effects of Dox or rtTA expression (Fig. 2J, fig. S2D, and movie S1). This cystic spheroid phenotype was gradually lost after Dox withdrawal (fig. S2E), showing the reversible nature of the reprogramming, similar to the later recovery stage of injury response in vivo (*28*). Consistent with the acquisition of the “fetal-like gene signature” by partial reprogramming of the intestine in vivo (Fig. 2, D and E), cystic spheroids resulting from OSKM induction resembled spheroids established from fetal mouse intestine (fig. S2F): They were positive for Sca1 and Trop2 (Fig. 2K and fig. S2G) and expressed the fetal genes *Ly6a* (encoding Sca1), *Anxa1*, and *Tacstd2* (encoding Trop2) in a dose-dependent manner (fig. S2H). This was clearly correlated with active YAP signal (Fig. 2L) and expression of the Yap downstream target gene, *Ccn2* as well as the accompanied key transcription factor, *Tead4* (fig. S2I). It is also noteworthy that Yap activation with XMU-MP-1, Mst1/2 inhibitor, induced cystic spheroid formation with clear active Yap signal (Fig. 2M). Consistently, bulk RNA-seq using organoids also showed that OSKM induction (fig. S2J) led to the enrichment of the fetal and Yap signatures (Fig. 2N and table S1).

### Generation of two distinct injury-responsive–like cells by partial reprogramming

**Fig. 3.**
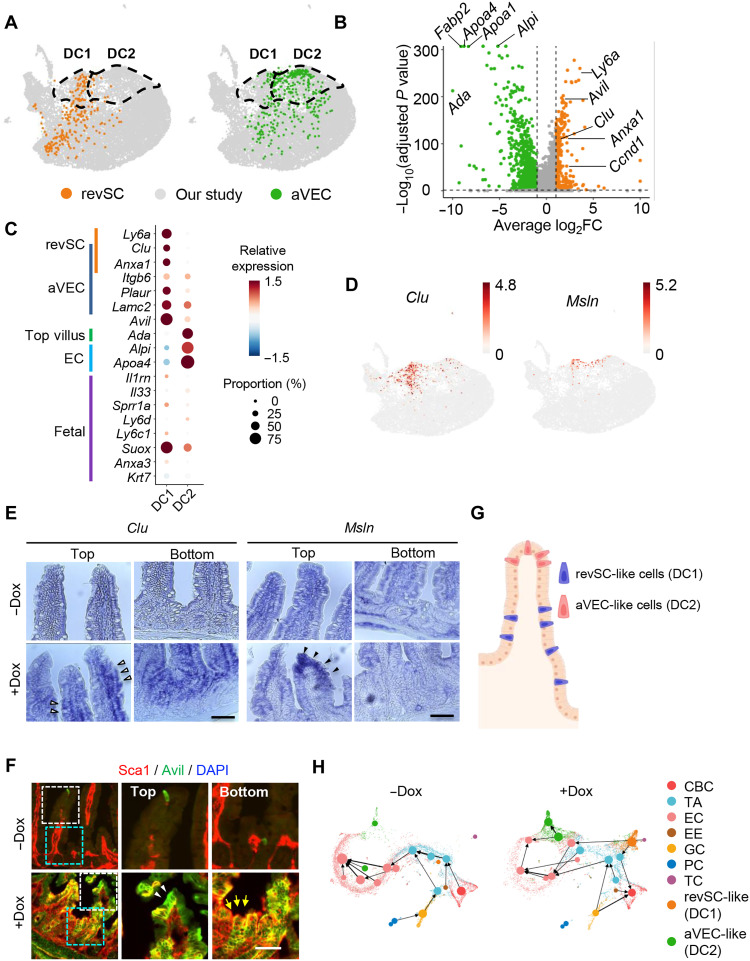
Generation of two distinct injury-responsive–like cells by partial reprogramming. (**A**) Projection of revSC (left) and aVEC (right) cells onto our scRNA-seq data. Cells in our study are indicated in gray. (**B**) Volcano plot showing differentially expressed genes (DEGs) between DC1 (right) and DC2 (left). (**C**) Dot plot for relative expression of marker genes of revSC, aVEC, top-villus, EC, and fetal signature genes in DC1 and DC2. (**D**) UMAP plots showing gene expression of *Clu* and *Msln*. (**E**) In situ hybridization (ISH) of *Clu* and *Msln* in the intestine of iOSKM mice. White arrowhead indicates *Clu*^+^ revSC-like cells, while black arrowhead indicates *Msln*^+^ aVEC-like cells. (**F**) IF of Sca1 and Avil in the intestine of iOSKM mice. White arrowhead indicates Sca1^−^Avil^+^ aVEC-like cells, while yellow arrow indicates Sca1^+^Avli^+^ revSC-like cells. (**G**) Graphical presentation of spatial presence of revSC (DC1) or aVEC (DC2)- like cells. (**H**) PAGA graphs showing velocity-directed arrows from cluster to cluster in −Dox (left) and +Dox (right). Scale bar, 50 μm.

Previous studies demonstrated that two distinct populations, revSCs and aVECs, appear via (de)differentiation in the crypt and villi, respectively, upon injury and repair the damage (*8*, *9*). To examine the characteristics of DC1 and DC2, the newly formed clusters with fetal gene signatures we identified after partial reprogramming (Fig. 2, B and D), the transcriptome profiles of DC1 and DC2 were compared with those from revSCs induced by crypt damage with IR (Fig. 3A and fig. S3A) and aVECs induced by villi atrophy (Fig. 3A and fig. S3B). The DC1 subset was characterized by high expression of revSC markers (87 genes including *Clu*, *Anxa1*, *Ccnd1*, and *Ly6a*) (Fig. 3B and fig. S3, C and D), while the DC2 subset was distinguished from DC1 by mature EC markers highly expressed at the top of villi (353 genes including *Ada*, *Fabp2*, *Apoa4*, *Apoa1*, and *Alpi*) (Fig. 3B and fig. S3, C and E). Among reported aVEC markers, *Clu* and *Anxa1* were mainly found in DC1 (Fig. 3C), while *Itgb6*, *Plaur* (Fig. 3C), *Cldn4*, *Lamc2* (fig. S3F), as well as *Msln* (Fig. 3D), were expressed in both DC1 and DC2 likely because of existence of overlapping makers for revSC and aVEC (fig. S3G). These results suggest that DC1 and DC2 considerably share molecular features with injury-induced revSC and aVEC, respectively.

Subsequent RNA in situ hybridization revealed that *Clu*, exclusively expressed in DC1 (Fig. 3D), was mainly elevated in the middle to the bottom of the villi as well as in the crypts (Fig. 3E, left), while *Msln*, expressed in DC1 and DC2 (Fig. 3D), was elevated at the top of the villi (Fig. 3E, right). It was consistent with the result that top villus genes, including *Ada* (*29*) were enriched in DC2, not DC1 (fig. S3, E and H). The discrete positions of DC1 and DC2 (Fig. 3E) were also confirmed by immunostaining with Sca1 (encoded by *Ly6a* expressed in DC1) and Avil (expressed in both DC1 and DC2) (Fig. 3F and fig. S3I). The Sca1^−^Avil^+^ population resides at the top of the villi, representing DC2, while double-positive cells, representing DC1, are mostly found in the bottom villi as well as crypts (Fig. 3, F and G). As expected, our trajectory analyses positioned DC1 and DC2 close to TA/CBC and EC, respectively (Fig. 3H and fig. S3J). Accordingly, we defined the DC1 and DC2, populations produced by partial OSKM induction even in the absence of physical injury, as revSC-like and aVEC-like cells, respectively.

### Promotion of intestinal repair by partial reprogramming

**Fig. 4.**
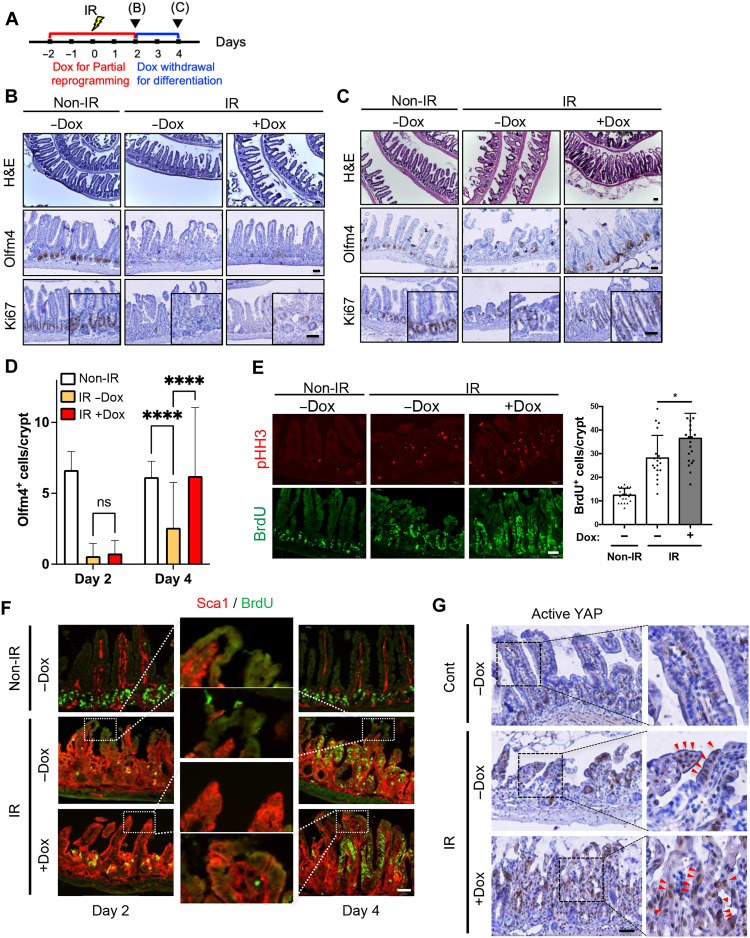
Promotion of intestinal repair by partial reprogramming. (**A**) Experimental scheme for 10-Gy IR and Dox treatment in iOSKM mice. The day after IR was indicated. Dox was treated 2 days before IR for 4 days and then removed to produce differentiated cells from OSKM-induced dedifferentiated cells for regeneration. Analysis at 2 and 4 dpi were showed in (B) and (C), respectively. (**B** to **D**) H&E histology and IHC of Olfm4 and Ki67 in the intestine of iOSKM mice after IR at 2 dpi (B) and at 4 dpi (C) with the quantification of Olfm4^+^ cells per crypt (*n* = 42 in total 3 mice of each condition) (D). (**E**) IF of phospho-histone H3(pHH3) and BrdU in the intestine of iOSKM mice after IR at 4 dpi (left) and quantification of BrdU+ cells per crypt (−Dox and non-IR, *n* = 20; −Dox and IR, *n* = 19; +Dox and IR, *n* = 22 in total 3 mice of each condition) (right). (**F**) IF of Sca1 and BrdU in the intestine of iOSKM mice after IR at 2 and 4 dpi. Top of the villi were magnified. (**G**) IHC of Active YAP in the intestine of iOSKM mice after IR at 4 dpi. Red arrowheads indicate active YAP. Data represent the mean with SD. Student’s *t* test: *P < 0.05, ****P < 0.0001. Scale bar, 50 μm.

The production of OSKM-induced injury-responsive**–**like cells led us to hypothesize that partial reprogramming may assist intestinal regeneration after injury. Because Oct4 expression was evident by 3 days after Dox administration (fig. S4A), “reprogrammable mice” were pretreated with Dox 2 days before IR. The intestines were harvested on day 2 and day 4 after IR-induced injury to examine the effect of partial reprogramming on acute tissue damage and the subsequent repair process (Fig. 4A). On day 2 after IR, clear depletion of Olfm4 and Ki67 signals in the crypt was observed regardless of Dox treatment (Fig. 4B), implying that similar crypt damage had occurred. In sharp contrast, on day 4, Dox-treated mice exhibited complete epithelial repair, along with re-expression of Olfm4 and Ki67 in the crypt and the TA zone, while mice without Dox treatment did not (Fig. 4C and fig. S4B). These observations correlated with the level of Olfm4+ cells in the crypts day 2 and day 4 after IR injury (Fig. 4D). This accelerated repair induced by OSKM expression was associated with a significant increase in proliferating cells in the TA zone [pulse-chased with 5-bromo-2′-deoxyuridine (BrdU) incorporation] (Fig. 4E), Trop2-positive fetal-like cells (fig. S4C), and robust Sca1 expression throughout the entire villi (Fig. 4F) at 4 days after IR, along with increased active Yap signal (Fig. 4G). In particular, the intestinal damage by 5-fluorouracil (5-FU) is one of the common complications for chemotherapy (*30*). Upon the intestinal injury induced by 5-FU treatment to the mice (fig. S4D), the proliferating cells (determined by KI67) as well as Olfm4-positive cells were manifested in mice with OSKM induction (fig. S4E), along with Sca1 expression and BrdU-positive populations at day 4 (fig. S4F).

Despite the comparable caspase activity regardless of OSKM expression in the intestinal organoids 24 hours after IR (fig. S4G), which represented the comparable shrinkage (i.e., loss of buds) until 4 days post-irradiation (dpi), a significant acceleration in regeneration of organoids occurred in the Dox-treated organoids (fig. S4H). The enhanced recovery of the organoids by OSKM expression was highlighted by in vitro culture longer period of time (fig. S4I).

### Enhanced epithelial prostaglandin E2 synthesis

**Fig. 5.**
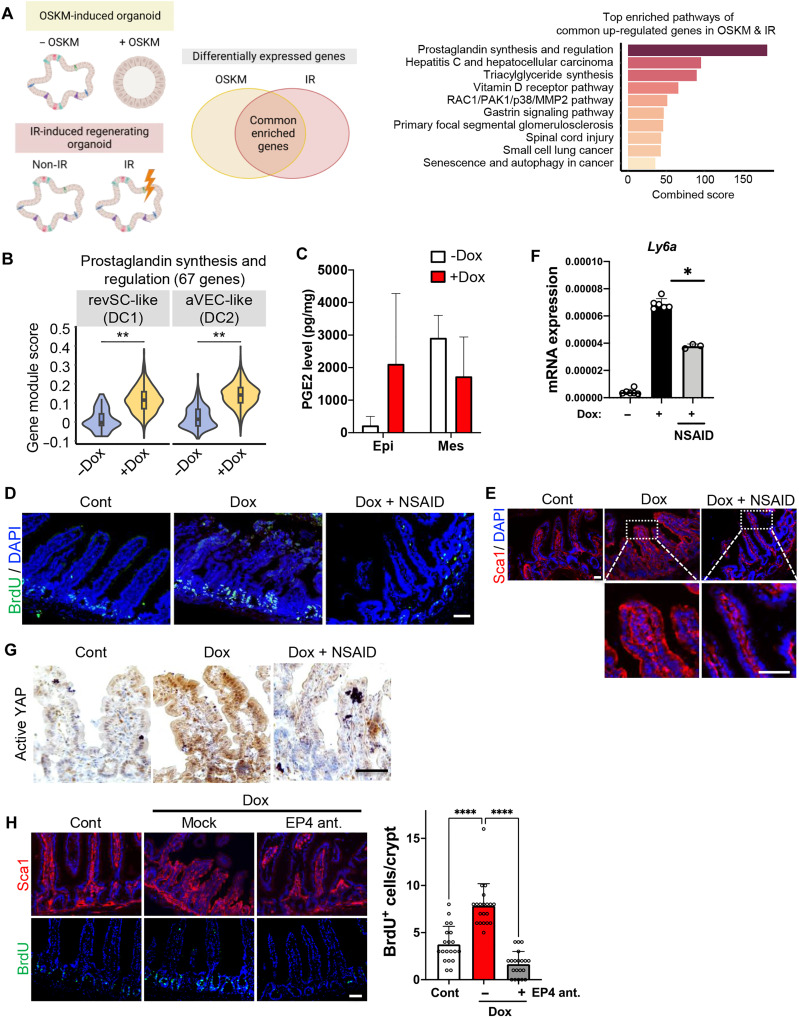
Enhanced epithelial PGE2 synthesis. (**A**) Experimental scheme for RNA-seq for OSKM- or IR-induced regenerating intestinal organoids (left), and a bar plot showing enrichR combined scores of top 10 commonly up-regulated pathways in OSKM-, IR-induced regenerating intestinal organoids compared to their control intestinal organoids (right). (**B**) A violin plot showing “Prostaglandin synthesis and Regulation” signature score in cell types (**C**) PGE2 level (pg/mg) of mouse intestine in epithelium (Epi) and mesenchyme (Mes) with or without Dox treatment. (**D**) IF of BrdU in the intestine of iOSKM mice, Cont (left), Dox (Dox-treated, middle), and Dox + NSAID (Dox- and dexibuprofen-treated, right). (**E**) IF of fetal markers, Sca1, in iOSKM mice intestine treated with Dox and NSAID for 4 days. DAPI for nuclear staining. (**F**) mRNA expressions of *Ly6a* in intestinal epithelial cells of iOSKM mice. *n* = 6; 2 mice × 3 technical replicates. (**G**) IHC of Active YAP in the intestine of iOSKM mice after Dox and NSAID administration for 4 days. (**H**) IF of Sca1 and BrdU in the intestine of iOSKM mice, Cont (left), Dox (Dox-treated, middle), and Dox + Ep4 ant (Dox and GW627368 treated, right) and quantification of BrdU+ cells per crypt. Data represent the mean with SD. Student’s *t* test: *P < 0.05, **P < 0.01, and ****P < 0.0001. Scale bar, 50 μm.

Partial reprogramming produced injury-responsive**–**like cell populations (Fig. 3) along with the acquisition of the fetal gene signature (Fig. 2), similar to injury-induced dedifferentiation; thus, a common molecular mechanism would exist between partial reprogramming and injury-induced dedifferentiation (Fig. 5A). To this end, we examined the commonly altered gene expression profiles of organoids after partial reprogramming and IR-mediated injury and found that a “Prostaglandin synthesis and Regulation” signature was apparently most enriched (Fig. 5A). The enrichment of the prostaglandin signature was commonly manifested in organoid from IR-mediated injury, fetal intestine, and partial reprogramming (fig. S5A). Consistently, significant induction of the prostaglandin signature was observed in DC1 and DC2 populations upon Dox treatment (Fig. 5B and table S1). Notably, prostaglandins E2 (PGE2), naturally produced by cyclooxygenase 1 (Cox1) and Cox2 (encoded by *Ptgs1* and *Ptgs2*, respectively), have been extensively studied in tissue regeneration (*31*). It is also known that inhibition of PGE2 degradation promotes tissue regeneration of bone marrow, colon, and liver (*32*) and aged muscle (*33*). While the natural epithelial regeneration of intestine is promoted by paracrine effect of PGE2 from mesenchymal *Ptgs2* expression (*18*, *34*, *35*), increased PGE2 production was observed from intestinal epithelium after OSKM induction but not from mesenchyme (Fig. 5C).

When treated with a nonsteroidal anti-inflammatory drug (NSAID), which is an inhibitor of Cox1 and Cox2, blocking the synthesis of PGE2, fetal-like transition by partial reprogramming was compromised. The oral administration of dexibuprofen, a typical NSAID, reverted the effect of partial reprogramming, such as an increase of BrdU-positive population in crypt (Fig. 5D) and Sca1-positive population (Fig. 5E), corresponding to the level of *Ly6a* expression (Fig. 5F). Consistently, fetal-like phenotypes such as spheroid formation and Sca1 expression were reversed in the organoid model by NSAID treatment (fig. S5, B to D). We also observed that YAP activation in the intestine (Fig. 5G) and intestinal organoid (fig. S5E) by partial reprogramming was also markedly attenuated by NSAID treatment.

To ascertain the downstream receptor responsible for the effects of PGE2, we assessed the expression levels of PGE2 receptor (EP) subtypes 1, 2, 3, and 4 in the small intestine. Notably, Prostaglandin E receptor 4 (*Ptger4*), encoding EP4, exhibited significantly higher expression in the intestinal epithelium compared to the other receptors (fig. S5F) and was only marginally increased in response to OSKM expression (fig. S5G). The marked attenuation of Sca1 expression and a reduction in the number of progenitors by simultaneous inhibition of EP4 using the antagonist GW627368 (Fig. 5H) highlighted the significance of PGE2 and EP4 axis for intestinal regeneration. Our findings were corroborated through gene set enrichment analysis (GSEA) using two different gene sets: up-regulated genes by PGE2 treatment in human colonic epithelial organoids (*34*) and down-regulated genes in conditional EP4 knockout mice (*35*). GSEA revealed significant enrichment of both gene sets in OSKM-induced intestinal organoids (fig. S5, H and I, and table S1). These results underscore the pivotal role of the PEG2-EP4 axis in facilitating injury-free regeneration in response to OSKM.

### Cox1 activity for partial reprogramming-induced regeneration

**Fig. 6.**
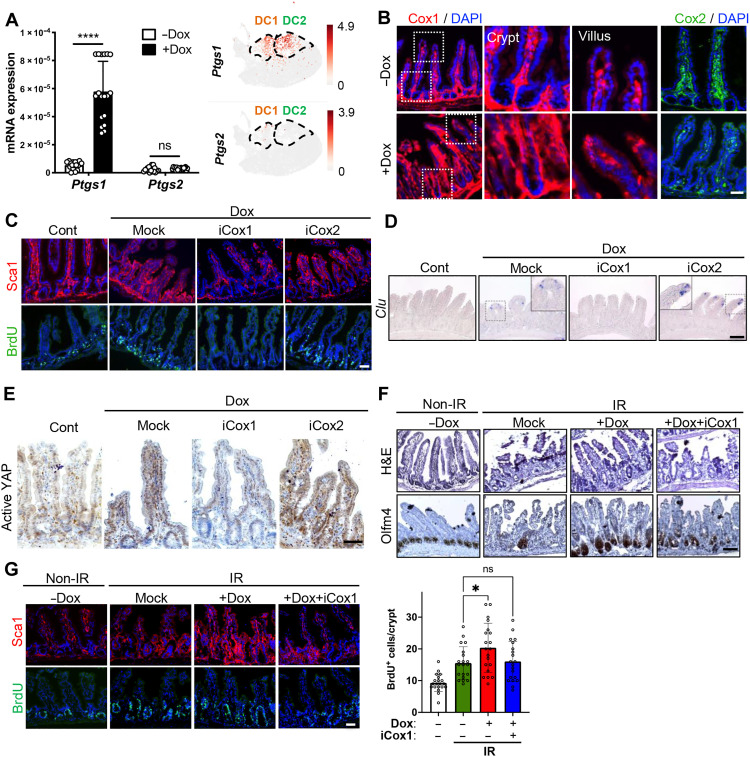
Cox1 activity for partial reprogramming-induced regeneration. (**A**) mRNA expressions of *Ptgs1* and *Ptgs2* in the intestinal epithelial cells of iOSKM mice. *n* = 18; 6 mice × 3 technical replicates (left). UMAP plots showing gene expression of *Ptgs1* and *Ptgs2* (right). (**B**) IF of Cox1 (left) and Cox2 (right) in the intestine of iOSKM mice 4 days after Dox administration. DAPI for nuclear staining. (**C**) IF of Sca1 and BrdU in the intestine of iOSKM mice. Dox was treated for 4 days with Cox1 inhibitor (iCox1: SC-560) or Cox2 inhibitor (iCox2: celecoxib) treatment for 2 days. DAPI for nuclear staining. (**D**) In situ hybridization of *Clu* in the intestine of iOSKM mice. (**E**) IHC of Active YAP in the intestine of iOSKM mice. (**F**) H&E histology and IHC of Olfm4 in the intestine of iOSKM mice at 4 dpi. Dox for 4 days and Cox1 inhibitor for 2 days were treated. (**G**) IF of Sca1 and BrdU in the intestine of iOSKM mice (left) and quantification of BrdU-positive cells per crypt (*n* = 20 in total three mice for each condition) (right). Data represent the mean with SD. Student’s *t* test: *P < 0.05 and ****P < 0.0001. Scale bar, 50 μm.

The diminished YAP activity by NSAID (Fig. 5G), prompted us to examine Cox1 and Cox2 more closely, because PGE2, of which production was manifested in intestinal epithelium by OSKM induction (Fig. 5C), is known to activate YAP through EP4 (*36*). Quantitative real-time polymerase chain reaction (RT-PCR) data clearly revealed that *Ptgs1*, but not *Ptgs2*, was markedly induced by partial reprogramming (Fig. 6A, left). scRNA-seq data also confirmed that *Ptgs1* expression was specifically up-regulated in OSKM-induced clusters, DC1 and DC2 (Fig. 6A, right). Cox1 expression, normally occurring in Dclk1-positive tuft cells (*37*), was shown to be induced predominantly in Dclk1-negative epithelial cells (fig. S6A), in both villus and crypt by OSKM induction unlike Cox2 (Fig. 6B). Furthermore, the exclusive epithelial induction of *Ptgs1* by OSKM was evident through costaining with E-cadherin, an epithelial cell marker, in the intestine (fig. S6B) and also through the expression in intestinal organoids (fig. S6, C and D). Notably, in fetal organoids, the expression of Cox1, rather than Cox2, was significantly higher compared to adult organoids (fig. S6, E and F). This observation aligns with the notion that OSKM-induced regeneration exhibits a fetal-like phenotype. It is noteworthy that *Ptgs1* expression normally remains constant, while *Ptgs2* is highly inducible under tissue damage and causes an inflammatory response (*38*). To determine the functionality of Cox1 induction (not Cox2) in the intestinal epithelium by partial reprogramming, we used selective inhibitors of Cox1 and Cox2: SC-560, Cox1 inhibitor (iCox1); celecoxib, Cox2 inhibitor (iCox2). As predicted, inhibition of Cox1, but not Cox2, distinctively negated the effect of partial reprogramming, such as Sca1 expression, an increase of cryptic BrdU-positive population (Fig. 6C), formation of *Clu*-positive population (Fig. 6D), and YAP activation (Fig. 6E). Inhibition of epithelial Sca1 induction by Cox1 inhibitor was demonstrated in intestinal organoids (fig. S6G). After IR injury, intestinal regeneration and concurrent formation of Olfm4-positive cells at crypts by OSKM induction were markedly attenuated by treatment of Cox1 inhibitor (Fig. 6F), which was corresponding to the level of BrdU-positive population at crypt (Fig. 6G). Together, autonomous epithelial production of PGE2 by Cox1 expression is a key molecular event for the induced dedifferentiation with partial reprogramming.

Given the established roles of Myc in promoting cell proliferation (*39*), absence of Myc to achieve the effect of partial reprogramming would be desirable as described previously (*14*). Unlike trivial effect of Myc induction in the Dox inducible Myc (iMyc) organoids (fig. S7, A and B), induction of Oct4, Sox2, and Klf4 (i.e., OSK) in the Dox inducible OSK (iOSK) organoids (fig. S7C) was sufficient to trigger fetal-like spheroid formation (fig. S7D), promote fetal gene expression (fig. S7E) and induce *Ptgs1* expression (fig. S7F). These data underscore the dispensability of Myc for the partial reprogramming in the intestine.

## DISCUSSION

Despite multiple recent studies revealing regeneration induced by OSKM in diverse tissues (*14*–*17*), the phenotypic and mechanistic similarities between forced dedifferentiation and injury response-mediated (de)differentiation (*8*, *9*) remain undetermined. Here, on the basis of scRNA-seq analysis, we demonstrated that the distinct epithelial populations generated by OSKM-mediated partial reprogramming are comparable to injury-responsive subpopulations that are responsible for acute damage-repair in the crypts and villi, respectively. This observation was supported by the expression of specific markers such as *Clu*, *Msln*, *Sca1*, and *Avil* and associated YAP activation and expression of fetal genes such as *Trop2* and *Sca1*. Induction of OSKM in organoids produced a fetal intestinal organoid phenotype (spheroids) with the expression of fetal gene signatures and activation of Yap. The induction of two distinct populations, DC1 and DC2, by OSKM expression mimics the generation of two injury-responsive cell types, revSC and aVEC, respectively. This means that a single cause (i.e., OSKM) can faithfully recapitulate the injury-mediated tissue dedifferentiation in the intestinal epithelium.

Notably, tissue damage induces *Ptgs2* to promote PGE2 production not only for inflammatory response but also for damage repair (or regeneration) (*18*) through “adaptive cellular response to produce wound repair cells” (*34*). In a similar manner, we showed that OSKM-mediated partial reprogramming, associated with fetal-transition by OSKM, was the result of an aberrant induction of *Ptgs1* and prostaglandin synthesis. These findings suggest that OSKM-induced (de)differentiation (via induction of epithelial *Ptgs1*) would share the same molecular mechanism (i.e., prostaglandin biosynthesis and subsequent Yap activation) as injury response-mediated (de)differentiation (through mesenchymal *Ptgs2* induction). It is noteworthy that there is emerging evidence that PGE2 is critical for tissue repair (or regeneration) of not only the intestine (*18*, *34*) but also the skeletal muscle (*40*), kidney (*41*), and heart (*42*). Consistently, the inhibition of PGE2 degradation (*32*) promotes both tissue regeneration and rejuvenation (*33*), as demonstrated in a mouse model by OSKM-induced partial reprogramming (*15*, *17*, *21*).

Unlike the formation of blastema by injury-induced dedifferentiation for appendage regeneration in amphibians or fish, only limited dedifferentiation upon injury occurs in mammals. Nevertheless, the genomic profile of blastema formation and the subsequent appendage is closely associated with prostaglandin biosynthesis and blocked by NSAID treatment, suggesting that prostaglandin synthesis is evolutionarily conserved for injury-induced dedifferentiation (*43*). Notable is the fact that not only *Ptgs2* but also *Ptgs1* is significantly induced alongside blastema formation in reptiles (*44*). Thus, OSKM-mediated dedifferentiation would be accounted for by the anomalous induction of *Ptgs1* by partial reprogramming, possibly via epigenetic alterations. As a follow-up study, it would be of interest to determine the epigenetic profile of *Ptgs1* to account for the OSKM-induced *Ptgs1* expression in light of the drastic epigenetic alterations induced by partial reprogramming (*21*).

## MATERIALS AND METHODS

### Mice

*Col1A1^TetO-OSKM^;ROSA26^rtTA^* mice were obtained from the Jackson Laboratory (no. 011004). *ROSA26^rtTA^* mice were generated from *Col1A1^TetO-OSKM^;ROSA26^rtTA^* mice. Age-matched mice between 2 and 4 months were used for all experiments. For OSKM induction, mice were treated with Dox (0.15 mg/ml) in drinking water containing 5% sucrose for 4 days. For BrdU pulse-chase experiment, BrdU (150 mg/kg) was intraperitoneally injected 3 hours before euthanasia. For injury experiments, mice were treated with 10-Gy x-ray radiation 2 days after Dox treatment and euthanized at indicated time points. 5-FU (100 mg/kg) was intraperitoneally injected 2 days after Dox treatment. For the inhibition of prostaglandin production while OSKM induction mice were treated with Dox (0.15 mg/ml) in drinking water containing 5% sucrose for 4 days and addition of dexibuprofen (0.6 mg/ml) in the last 2 days. For Cox-1 and Cox-2 inhibition, SC-560 (15 mg/kg per day) and celecoxib (18.75 mg/kg per day) were intraperitoneally injected respectively for the last 2 days of Dox administration. GW627368 (1 mg/kg per day) was also intraperitoneally injected for the last 2 days. These animal experiments were conducted under the permission of Seoul National University Institutional Animal Care and Use Committee (permission number: SNU-210326-5-4, SNU-221017-2).

### In vitro culture of intestinal organoid

For in vitro culture of adult organoids, mouse small intestine was washed with phosphate-buffered saline (PBS) and cut longitudinally. Villi were scraped away with a microscope slide, and the intestine was washed three times with cold PBS. The intestine was then incubated in gentle cell dissociation reagent (STEMCELL Technologies, no. 07174) for 15 min. The isolated crypts were embedded in Matrigel (Corning, 354234) and seeded on a 48-well plate. After polymerization, intesticult (STEMCELL Technologies, no. 06005) containing gentamicin (50 μg/ml) was added and refreshed every 2 days. For passaging, the organoids were suspended in cold Dulbecco’s modified Eagle’s medium (DMEM)/F12 (Gibco) and were embedded in fresh Matrigel and seeded on plate followed by addition of culture medium. For OSKM induction, organoids were treated with Dox (0.1 or 0.5 μg/ml) for 3 days. For injury experiments, organoids were subjected to 10-Gy x-ray radiation, both with and without the presence of Dox. The organoids were passaged on day 7 and day 14 after IR. For monitoring the budding growth of individual organoid after IR, the irradiated organoids were maintained until day 8 after IR without passage. For Dox-inducible expression of Myc, PB-TET-Myc plasmid (Addgene, no. 20888) was used. For Dox-inducible expression of OSK (Oct4, Sox2, and Myc), we generated TET-OSK with piggyBac transposon (PB-TET-OSK). Myc and F2A sequence-deleted fragments were generated by PCR using PB-TET-MKOS plasmid (Addgene, no. 20959) and linked by In-Fusion cloning. PB-TET-Myc or PB-TET-OSK were electroporated into rtTA intestinal organoids following single cell dissociation, and then selected by G418 (400 μg/ml) treatment. For the inhibition of prostaglandin production while OSKM induction, organoids were treated with Dox (0.5 μg/ml) alone for 1 day and with 3 μM dexibuprofen addition for 2 days. For the inhibition of Cox-1 and Cox-2, organoids were treated with 3 μM celecoxib and 0.675 μM SC-560, respectively. For in vitro culture of fetal organoids, fetal small intestines were cut into small pieces and then dissociated with gentle cell dissociation reagent (STEMCELL Technologies) for 15 min. The isolated epithelial units were embedded in Matrigel and maintained in conditions identical to those used for adult intestinal organoids.

### Immunohistochemistry and immunofluorescence

For intestine sections, Swiss rolls of mouse intestines were fixed in 4% paraformaldehyde (PFA) at 4°C overnight, immersed in 30% sucrose at 4°C overnight, and then cryopreserved in Tissue-Tek optimal cutting temperature (OCT) compound (Sakura Finetek). For organoid sections, intestinal organoids were fixed in 4% PFA at room temperature for 15 min, immersed in 30% sucrose at 4°C overnight, and then cryopreserved in OCT compound. For immunohistochemistry, 6-μm sections were treated with 3% H_2_O_2_ in methanol for blocking endogenous peroxidase activity. For antigen retrieval. Slides were kept in tris-EDTA buffer (pH 9.0) at 95°C for 20 min. Nonspecific binding was prevented by incubation with 10% fetal bovine serum (FBS) in PBS for 1 hour. Slides were incubated with a primary antibody at 4°C overnight or at room temperature for 1 hour. Then, the slides were incubated with a horseradish peroxidase–conjugated secondary antibody at room temperature for 2 hours, followed by 5- to 10-min incubation with 3,3′-diaminobenzidine substrate (Vector Laboratories, SK-4100). Hematoxylin solution (Sigma-Aldrich, GHS3) was used for nuclear staining and then washed with tap water. Slides were covered with slide glass using MOWIOL solution. Samples were visualized with a microscope (Leica DM500). For immunofluorescence, sections were incubated with a primary antibody in 4% bovine serum albumin (BSA) in PBS at 4°C overnight or at room temperature for 1 hour. Then, the slides were incubated with Alexa Fluor secondary antibodies conjugated to 488 or 594 fluorophores (Invitrogen) at room temperature for 2 hours. 4′,6-Diamidino-2-phenylindole (DAPI) (Thermo Fisher Scientific) was used for nuclear staining and then washed with PBS-T. slides were covered with slide glass using MOWIOL solution. Fluorescence microscopy (Olympus) was used for imaging samples. Following primary antibodies were used for IHC and IF: mouse anti-2A peptide (Millipore, MABS2005, 1:200), rabbit anti-Active YAP1 (Abcam, ab205270, 1:1000), rabbit anti-Avil (Abcam, ab72210, 1:500), rabbit anti-BrdU (Novus Biologicals, NBP2-14890, 1:200), CD44 (BioLegend, 103015, 1:500), rabbit anti-Ki67 (Abcam, ab16667, 1:500), rabbit anti-Lysozyme (Abcam, ab108508, 1:200), mouse anti-Oct4 (BD Biosiences, 611203, 1:500), rabbit anti-Olfm4 (Cell Signaling Technology, 39141, 1:400), rabbit anti-phosphohistone H3 (Cell Signaling Technology, 53348, 1:1000), rat anti-Sca1 (BioLegend, 122501, 1:500), rabbit anti-Sox2 (Millipore, AB5603, 1:400), goat anti-Trop2 (R&D Systems, AF1122, 1:100), mouse anti-Cox1 (Santa Cruz Biotechnology, sc-19998, 1:100), mouse anti-Cox2 (Santa Cruz Biotechnology, sc-19999, 1:100), rabbit anti–E-cadherin (Cell Signaling Technology, 3195, 1:1000), and rabbit anti-Dclk1(Abcam, ab31704, 1:1000).

### Histology and AB-PAS staining

For histological analysis, sections were stained with hematoxylin and eosin, dehydrated, and then covered with slide glass using Canada balsam (Sigma-Aldrich). For staining of GCs, sections were stained using AB and PAS. Samples were visualized with a microscope (Leica DM500).

### In situ hybridization

Intestine were fixed overnight in 4% PFA in PBS. For whole-mount in situ hybridization, the intestine was treated with proteinase K (20 μg/ml) (AM2546, Thermo Fisher Scientific, USA) for 10 min at room temperature. Antisense RNA probes were labeled with digoxigenin (Roche, Switzerland). Digoxigenin (DIG)–labeled RNA probes were prewarmed at 85°C and hybridized to the intestine specimen overnight at 69°C. After whole-mount in situ hybridization, the specimens were cryo-sectioned at a thickness of 12 μm. At least 10 specimens were examined for each condition. The primer sequences of the Clu and Msln are as follows:

*Clu*- Forward: 5′-GAG ATT CAG AAC GCC GTC CA-3′.

*Clu*- Reverse: 5′-CTC TTG TGT GGG AAG CCG AT-3′.

*Msln*- Forward: 5′-GTG CCC ACT TCT TCT CCC TC-3′.

*Msln*- Reverse: 5′-GGT GCC ATC TAC ACA AGC CT-3′.

For *Lgr5* in situ hybridization, RNAscope 2.5 HD Assay – RED (ACD, 322360), RNAscope Probe- Mm-Lgr5 (ACD, 312171), and RNAscope H202 & Protease Plus Reagents (ACD, 322330) were used with mouse intestinal sections prepared as for immunofluorescence and immunohistochemistry, following provided protocol (Document Number 322360-USM).

### RNA isolation and quantitative RT-PCR

Easy-BLUE RNA isolation kit (iNtRON Biotechnology, no. 17061) is used for total RNA extraction. PrimeScript RT reagent kit (TaKaRa, RR036A) is used to generate cDNA from RNA extracted. Quantitative RT-PCR analysis was performed with Light Cycler-480II (Loche) using TB-Green (Takara, RR420) following the supplier’s instructions.

### Immunoblotting

Cell lysates were extracted with RIPA buffer supplemented with 1% protease inhibitor cocktail and 0.1% sodium orthovanadate. After 1 hour incubation on ice, total protein was extracted after centrifugation. The concentration of total protein was quantified by a bicinchoninic acid (BCA) protein assay kit (Thermo Fisher Scientific). Total protein (10 μg) was separated on 10% SDS–polyacrylamide gel electrophoresis. Separated protein in the gel was transferred to polyvinylidene difluoride membrane. Membrane with protein was blocked with 10% skim milk in tris-buffered saline containing 0.1% Tween 20 (TBS-T) for 1 hour and then washed three times by TBS-T. The membrane was incubated with primary antibody in TBS-T at 4°C overnight. Following primary antibodies were used: mouse anti-Oct4 (BD Biosiences, 611203, 1:1000), and mouse anti–α-tubulin (Santa Cruz Biotechnology, sc-8035, 1:1000). Incubated membrane was washed three times with TBS-T. The membrane was incubated at room temperature with horseradish peroxidase–conjugated secondary antibody in TBS-T for 1 hour, followed by wash with TBS-T three times. Immunoreactivity was detected by Chemi-Doc using WEST-Queen kit (iNtRON Biotechnology, no. 16026).

### Flow cytometry

Intestinal organoids were incubated in TrypLE (Invitrogen) for 15 min at 37°C for single-cell dissociation and then washed with PBS three times. For Sca1 staining, the dissociated cells were incubated in PE/Cy7-conjugated anti-Sca1 antibody (BD Biosciences, 558162, 1:100) in 4% BSA in PBS for 20 min. Then, the cells were washed three times and analyzed by a flow cytometer (BD LSRFortessa X-20). The data were analyzed with FlowJo software.

### Caspase-3 activity assay

Caspase-3 activity was detected using colorimetric assay kits (Abcam, ab39401). The kits were used according to the manufacturer’s protocols. Briefly, 24 hours after IR, organoids were lysed in the supplied lysis buffer for 10 min at 4°C. Supernatants were collected and incubated with the supplied reaction buffer containing dithiothreitol and DEVD-p-NA substrate at 37°C for 2 hours. The reactions were measured by changes in absorbance at 400 nm using a microplate reader.

### Mouse intestinal epithelial and mesenchymal cells isolation

Mouse intestine was dissected and washed with PBS and cut longitudinally. The intestine was incubated in PBS containing 2 mM EDTA for 30 min, followed by vigorous shaking and washed with PBS. For mesenchymal cells isolation, epithelial cells were removed as mentioned above, and incubated in DMEM containing 10% FBS, Collagenase XI (300 U/ml), Dispase I (2 U/ml) and DNase II (50 U/ml) for 1 hour, at 37°C. Isolated cells were washed with 2% sorbitol containing Red Blood Cell Lysis Solution.

### PGE2 assay

Epithelial and mesenchymal cells were isolated as mentioned above with every reagent containing indomethacin (6 μg/ml). Isolated cells were sonicated for 3 min using cycles of 2 s, followed by centrifugation for 5 min, 13,000 rpm. Using a PGE2 enzyme-linked immunosorbent assay kit (R&D Systems, no. KGE004B), PGE2 level was measured and standardized with protein level using BCA Protein Assay Kit (Thermo Fisher Scientific, no. 23227).

### scRNA-seq library preparation

To prepare single cells, whole epithelial segments were isolated with 2 mM EDTA for 30 min, washed with PBS, and then dissociated to single cells with TrypLE (Invitrogen) at 37°C for 30 min, with mixing every 10 min. Dissociated cells were filtered through a 40-μm cell strainer on ice and washed three times with cold DMEM/F12. Single-cell suspensions with 90% cell viability were processed on the 10x Chromium Controller using Chromium Next GEM Single Cell 3’ Reagent Kits v3.1 (10x Genomics) according to the manufacturer’s instructions. Cells were partitioned into nanoliter-scale Gel Beads-in-Emulsion (GEMs) with target recovery of 10,000 cells. The single-cell 3' mRNA seq library was generated by reverse transcription, cDNA amplification, fragmentation, and ligation with adapters followed by sample index PCR. Resulting libraries were quality checked by Bioanalyzer and sequenced on an Illumina NovaSeq 6000 (index = 8 bases, read 1 = 26 bases, and read 2 = 91 bases).

### scRNA-seq data preprocessing

We processed raw FASTQ files for scRNA-seq using the CellRanger software suite (v5.0.1) (*45*). Reads were mapped to the mouse reference genome (GRCm38) with the Ensembl GRCm38.102 GTF file. We also eliminated technical artifacts using the remove-background function of the CellBender (v0.2.0) python package (*46*). We excluded low-quality cells with <2.0 log_10_-scaled counts of UMIs and <50% of UMIs assigned to mitochondrial genes using the calculateQCMetric function of the scater (v1.22.0) R package (*47*). To remove cell-specific biases, cells were grouped using the quickCluster function of the scran (v1.22.0) R package (*48*) with default options and cell-specific size factors were calculated using the computeSumFactors function of the same package with default options. Raw counts of each cell were divided by cell-specific size factor and log_2_-transformed with the pseudocount of 1. To define highly variable genes (HVGs), we modeled the variance of the log-expression profiles of each gene and decomposed it into technical and biological components based on a fitted mean-variance trend using the modelGeneVar function of the scran R package. Genes with <0.05 false discovery rate (FDR) were defined as HVGs. For downstream analysis, we calculated the top 20 principal components (PCs) calculated from the normalized count matrix of HVGs. To remove technical effects between different samples, calculated principal components were corrected using the RunHarmony function of the harmony (v0.1.0) R package (*49*). A shared nearest neighbor (SNN) graph was constructed using the FindNeighbors function of the Seurat (v4.0.5) R package (*50*) with the top 20 PCs and cells were clustered using the FindClusters function with the default options based on a SNN graph. Cells were visualized on the two-dimensional UMAP plot using the RunUMAP function of the same package as with the first 20 PCs. Each cluster was defined as one of the major cell types based on the expression of canonical cell-type marker genes while clusters identified as nonepithelial cells—including *Ptprc* + immune cells—were excluded for downstream analysis. The remaining cells were regrouped and visualized on the 20 PCs using the same method described above. For projection, reference data (revSC and aVEC-containing scRNA-seq data, GSE123516, GSE169718, respectively) were preprocessed using the same methods as above. We annotated the major cell types mentioned in the paper based on the expression of canonical cell type–specific marker genes after excluding nonepithelial cells including *Ptprc* + immune cells. For our scRNA-seq and reference scRNA-seq data, differentially expressed genes (DEGs) of each cell type were computed using the FindMarkers function of Seurat R package.

### scRNA-seq data analysis

To infer the stemness of each cell, we estimated differentiation potency using the SCENT (v0.3.3) R package (*51*). A raw count matrix log-transformed with a pseudocount of 1.1 to avoid 0 values after log-transformation and used as an input matrix. Normalized data was integrated with the PPI network using the DoIntegPPI function and signaling entropy rate (SR) was computed using the CompSRana function with default options. In addition, we inferred differentiation potency using the CytoTRACE function of the CytoTRACE (v1.0.2) R package (*52*) with default options. For 1275 fetal signature genes (*24*) and 398 yap signature genes (*53*), gene signature score per cell was calculated using the AddModuleScore function of Seurat R package. All gene sets used for analysis are described in table S1. To project reference data (revSC and aVEC-containing scRNA-seq data) onto our data, for each reference data, we obtained k-Nearest Neighbors (k-NNs) from our data based on Pearson correlation coefficients of normalized expression data of HVGs between the reference data and our data using the knn.index.dist function of the KernelKnn (v1.1.4) R package (*54*). To visualize the projection results, for each reference data, we averaged two-dimensional coordinates of 10-NNs on the UMAP plot of our data. To infer the orientation of cellular differentiation, we performed RNA velocity analysis using the scVelo (v0.2.4) python package (*55*). For each condition, we generated the spliced and unspliced expression profiles using the run function of the velocyto (v0.17.17) python package (*56*) with the mouse reference masking GTF file (GRCm38) and the Ensembl GRCm38.102 GTF file. The raw profiles were natural log-transformed after excluding low expressed genes using the pp.filter_and_normalize function with default parameters except for min_shared_counts = 30. Moments for velocity estimation were computed using the pp.moments function with the default parameters except for n_neighbors = 5. RNA velocities were estimated using the tl.velocity function with the option of mode = dynamical. The velocity graphs were computed based on cosine similarities using the tl.velocity_graph function with default options. We visualized RNA velocity results on the two-dimensional t-SNE plot using the Palantir (v1.0.0) python package (*57*). For the Palantir t-SNE plot, we computed diffusion components (DCs) using the run_diffusion_maps function with the first 200 PCs. A k-Nearest Neighbor (kNN) graph (k = 30) was constructed from the first 20 DCs. The coordinated for t-SNE plot were computed using the run_tsne function with the options of perplexity = 300. To quantify the connectivity between the 19 cell clusters, we generated partition-based graph abstraction (PAGA) graph of each condition using the tl.paga function with default options. The PAGA graphs with velocity-directed edges were plotted using only directed arrows.

### Bulk RNA-seq library preparation

For the bulk RNA-seq library preparation of Dox-treated organoids, Total RNA was isolated from intestinal organoids using Easy-BLUE RNA isolation kit (iNtRON Biotechnology, no. 17061). One 1 μg of total RNA was processed for preparing mRNA sequencing library using MGIEasy RNA Directional Library Prep Kit (MGI) according to manufacturer’s instruction. The first step involves purifying the poly-A containing mRNA molecules using poly-T oligo attached magnetic beads. Following purification, the mRNA is fragmented into small pieces using divalent cations under elevated temperature. The cleaved RNA fragments are copied into first-strand cDNA using reverse transcriptase and random primers. Strand specificity is achieved in the RT directional buffer, followed by second-strand cDNA synthesis. These cDNA fragments then have the addition of a single “A” base and subsequent ligation of the adapter. The products are then purified and enriched with PCR to create the final cDNA library. The double-stranded library is quantified using QauntiFluor ONE dsDNA System (Promega). The library is circularized at 37°C for 30 min and then digested at 37°C for 30 min, followed by cleanup of circularization product. To make DNA nanoball (DNB), the library is incubated at 30°C for 25 min using DNB enzyme. Last, Library was quantified by QauntiFluor ssDNA System (Promega). Sequencing of the prepared DNB was conducted on the MGIseq system (MGI) with 100-bp paired-end reads. For the bulk RNA-seq library preparation of Fetal and irradiated organoids, total RNA was extracted with QIAzol reagent (catalog no. 79306; QIAGEN) according to the manufacturer’s instructions. The RNA-seq libraries were constructed using the NEXTflex Rapid Directional mRNA-seq kit (catalog no. NOVA-5138-11; PerkinElmer). Briefly, 1 μg of purified RNA was poly-A selected and fragmented with fragmentation enzyme. After first- and second-strand synthesis from a template of poly-A–selected/fragmented RNA, other procedures from adenylation to PCR amplification were done according to RNA-seq library construction steps. Sequencing was conducted on the Illumina NextSeq 500 with 75-bp single-end reads.

### Bulk RNA-seq processing and analysis

Low-quality bases and adapter sequences bases were trimmed using TrimGalore (www.bioinformatics.babraham.ac.uk/). The trimmed reads were aligned to the mouse genome assembly GRCm39 using STAR (v2.7.3a). Transcript abundance per gene such as expected read count or transcripts per million (TPM) was quantified by RSEM (v1.3.3) with mouse gene annotation GRCm39.104. Raw read counts were normalized by trimmed mean of M-values and log_2_ fold changes (log_2_FC) of genes between conditions were obtained by using the DESeq function of DESeq2 (v1.34.0) R package. Genes with log_2_FC > 0.5 and adjusted *P* < 0.05 were identified as differentially expressed genes (DEGs). Functional enrichment analysis of DEGs for “WikiPathway_2021_Human” database was performed using the enrichR (v3.1) R package. To build a yap signature, we selected 445 down-regulated genes by *Yap* knockout with log_2_FC < −2 from the table S1 in the previous study (*58*). To define gene signatures related to PGE2 signaling, we used one microarray data for human colonic epithelial organoids with or without PGE2 treatment (GSE116936) (*59*) and one RNA-seq data for WT and intestinal epithelial cell–specific EP4 knockout mice (GSE135859) (*60*). For GSE116936, raw read counts were preprocessed using the same methods as our bulk RNA-seq data. Genes with log_2_FC > 1 and adjusted *P* < 0.05 were selected as up-regulated genes. Mouse orthologous genes of the 128 up-regulated genes were found by using the getLDS function of the biomaRt (v2.50.3) R package (*61*) and used as “Upregulated genes by PGE2 treatment”. For GSE135859, average log_2_FC of each probe between conditions were calculated and 344 genes for probes with log_2_FC < −3 were used as “Downregulated genes by EP4 knockout”. With two curated gene signatures, GSEA on log_2_FC of iOSKM intestinal organoids compared to the control was performed using the fgsea function of the fgsea (v1.20.0) R package (*62*) with the options of nperm = 10,000. All gene sets used for analysis are described in table S1.

### Quantification and statistical analysis

The quantitative data are expressed as the mean values ± SD. Student’s unpaired *t* tests was performed to analyze the statistical significance of each response variable using the PRISM. *P* values less than 0.05 were considered statistically significant, *P < 0.05, **P < 0.01, ***P < 0.001, and ****P < 0.0001.
