# Supplementary material for: Partial in vivo reprogramming enables injury-free intestinal regeneration via autonomous Ptgs1 induction
Source: Sci Adv. 2023 Nov 24;9(47):eadi8454. doi: 10.1126/sciadv.adi8454 (PMC10672161; doi:10.1126/sciadv.adi8454)
Supplement: Supplementary file 1 — Figs. S1 to S7 Legend for table S1 Legend for movie S1 [file sciadv.adi8454_sm.pdf]

Science Advances
AAAS

Supplementary Materials for

Partial in vivo reprogramming enables injury-free intestinal regeneration via autonomous *Ptgs1* induction

Jumee Kim *et al.*

Corresponding author: Hyuk-Jin Cha, hjcha93@snu.ac.kr; Bon-Kyoung Koo, koobk@ibs.re.kr; Jong Kyoung Kim, blkimjk@postech.ac.kr

Sci. Adv. **9**, eadi8454 (2023)
DOI: 10.1126/sciadv.adi8454

The PDF file includes:

Figs. S1 to S7
Legend for table S1
Legend for movie S1

Other Supplementary Material for this manuscript includes the following:

Table S1
Movie S1

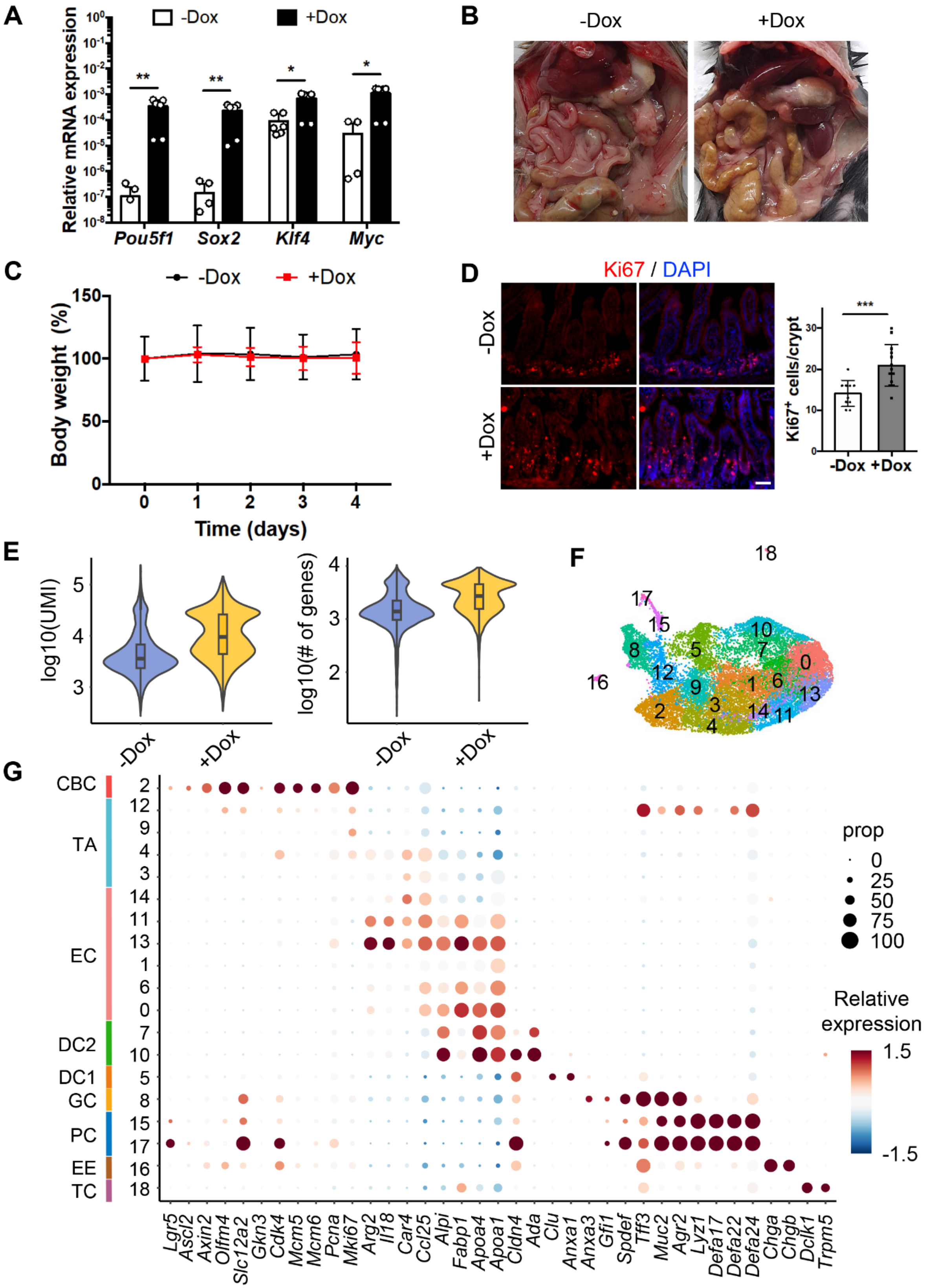
A
-Dox
+Dox
Relative mRNA expression
Pou5f1
Sox2
Klf4
Myc
B
-Dox
+Dox
C
-Dox
+Dox
Body weight (%)
Time (days)
D
Ki67 / DAPI
-Dox
+Dox
Ki67+ cells/crypt
E
log10(UMI)
log10(# of genes)
F
G
CBC
TA
EC
DC2
DC1
GC
PC
EE
TC
prop
Relative expression
Lgr5
Ascl2
Axin2
Olfm4
Slc12a2
Gkn3
Cdk4
Mcm5
Mcm6
Pcna
Mki67
Arg2
Il18
Car4
Ccl25
Alpi
Fabp1
Apoa4
Apoa1
Cldn4
Ada
Clu
Anxa1
Anxa3
Gfi1
Spdef
Tff3
Muc2
Agr2
Lyz1
Defa17
Defa22
Defa24
Chga
Chgb
Dclk1
Trpm5

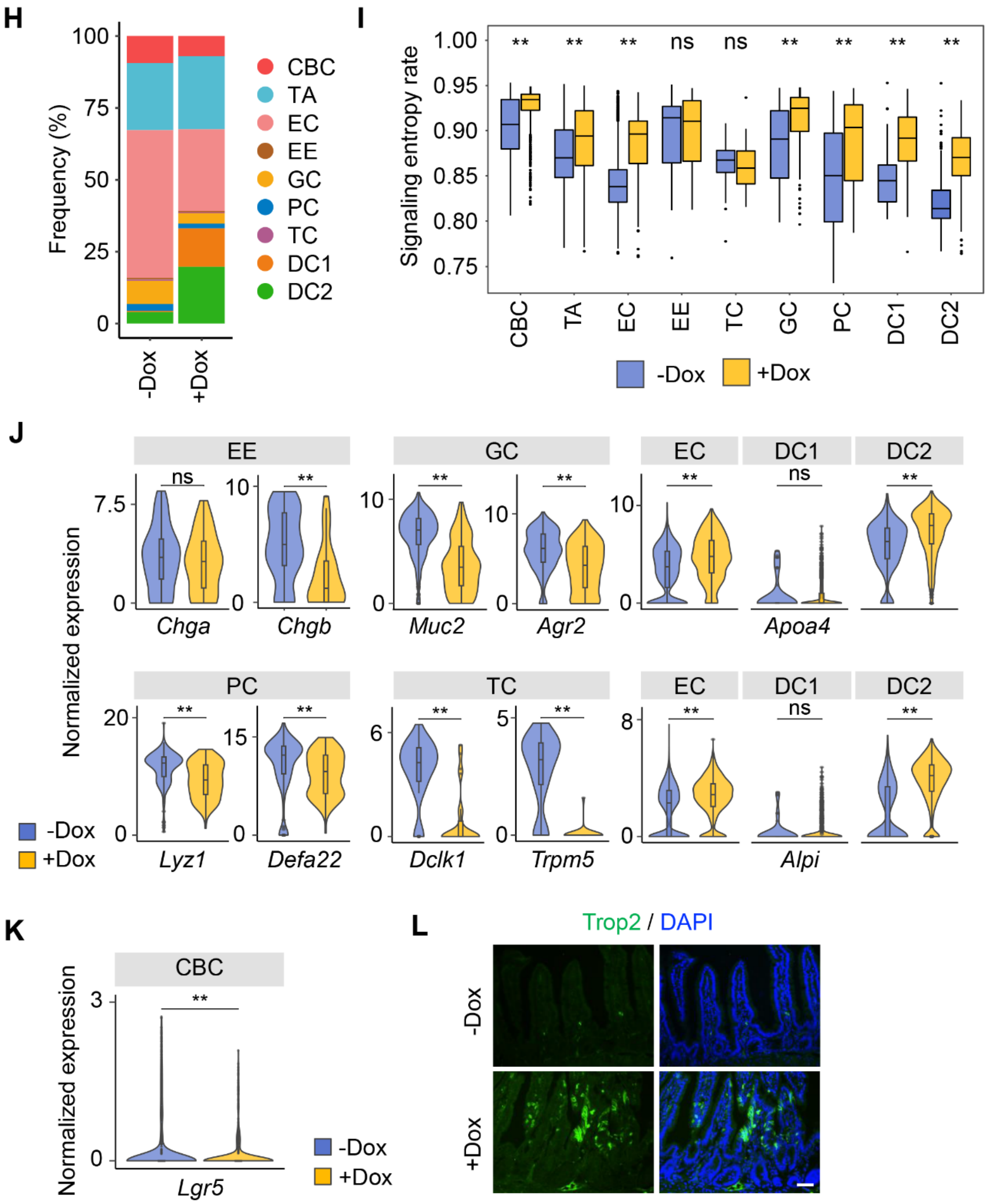

Fig. S1. Identification of dedifferentiated intestinal epithelial cells by partial reprogramming (**A**) Relative mRNA expression of OSKM in intestinal epithelial cells of iOSKM mice 4 days after Dox treatment. Data represent the mean with SD (n = 6; 3 mice x 2 technical replicates) (**B**) Necropsy of iOSKM mice 4 days after Dox treatment. (**C**) Body weight of iOSKM mice during Dox treatment. Data represent the mean with SD (n = 3 mice) (**D**) IF of Ki67 in the intestine (left)

and quantification of Ki67-positive cells per crypt (-Dox, $n = 10$; +Dox, $n = 13$) (right). (**E**) Violin plots showing log10-scaled value of unique molecular identifiers (UMIs) (left) and log10-scaled value of the number of features in –Dox and +Dox conditions. (**F**) UMAP plot of scRNA-seq from OSKM-induced mouse intestinal epithelium indicated with 18 distinct clusters. (**G**) Dot plot for expression of canonical marker genes per each cluster. (**H**) Bar plot showing cell type proportion in each condition. (**I**) Box plot indicating signaling entropy rate (SR) inferred by SCENT per each condition in different cell types. (**J**) Violin plots showing expression of differentiated cell-type marker genes between conditions in different cell types. (**K**) Violin Plot showing expression of *Lgr5* between conditions in CBC. (**L**) IF of Trop2 in the intestine of iOSKM mice. DAPI for nuclear staining. Data represent the mean with SD. Student's t-test: $p < 0.05$(*), $p < 0.01$(**), $p < 0.001$(***), ns, not significant. Scale bar = 50μm.

Figure S2

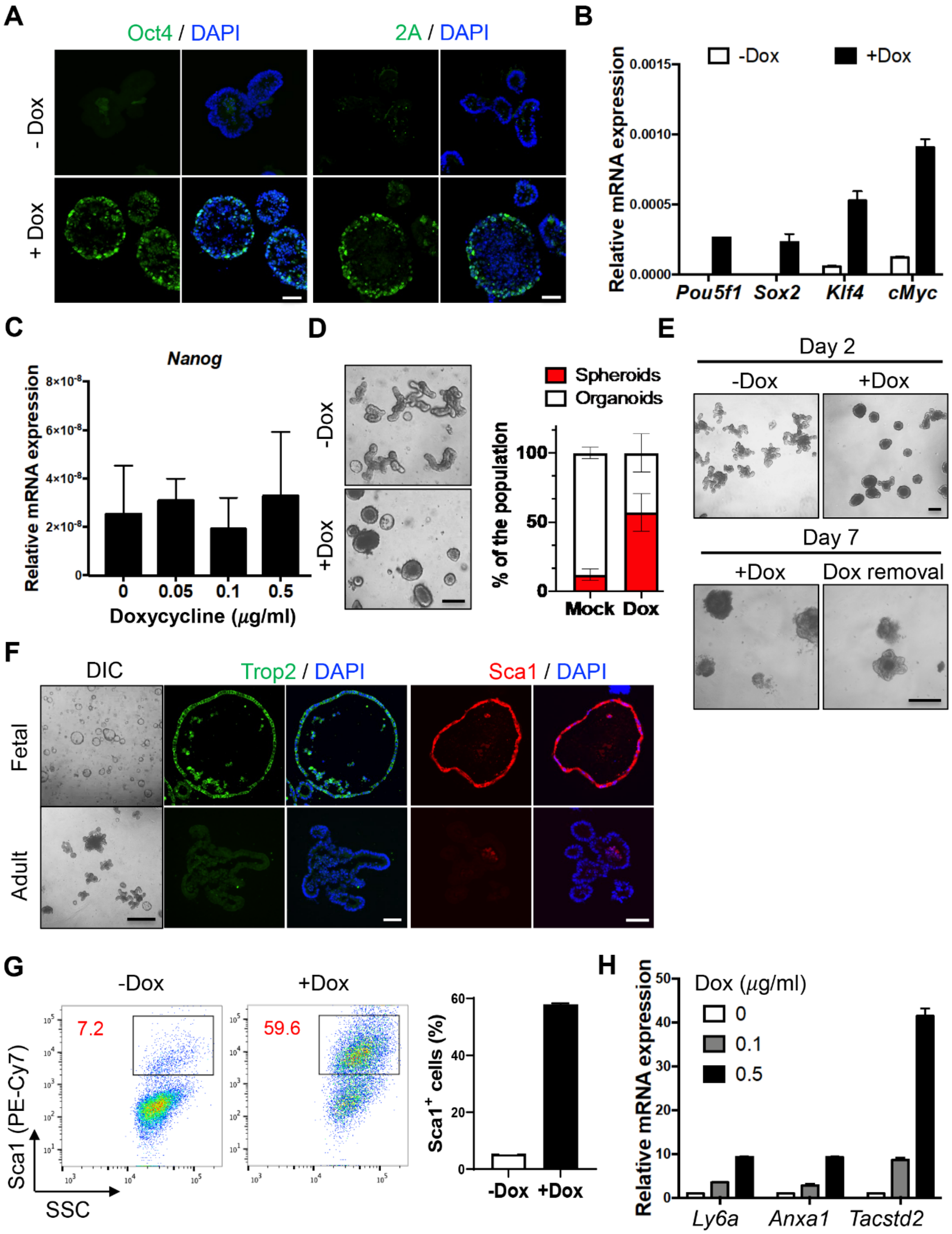
A
Oct4 / DAPI
2A / DAPI
- Dox
+ Dox
B
-Dox
+Dox
Relative mRNA expression
0.0015
0.0010
0.0005
0.0000
Pou5f1
Sox2
Klf4
cMyc
C
Nanog
Relative mRNA expression
Doxycycline (μg/ml)
0
0.05
0.1
0.5
D
-Dox
+Dox
Spheroids
Organoids
% of the population
100
50
0
Mock
Dox
E
Day 2
-Dox
+Dox
Day 7
+Dox
Dox removal
F
DIC
Trop2 / DAPI
Sca1 / DAPI
Fetal
Adult
G
-Dox
+Dox
7.2
59.6
Sca1 (PE-Cy7)
SSC
Sca1+ cells (%)
60
40
20
0
-Dox
+Dox
H
Dox (μg/ml)
0
0.1
0.5
Relative mRNA expression
50
40
30
20
10
0
Ly6a
Anxa1
Tacstd2

Figure S2

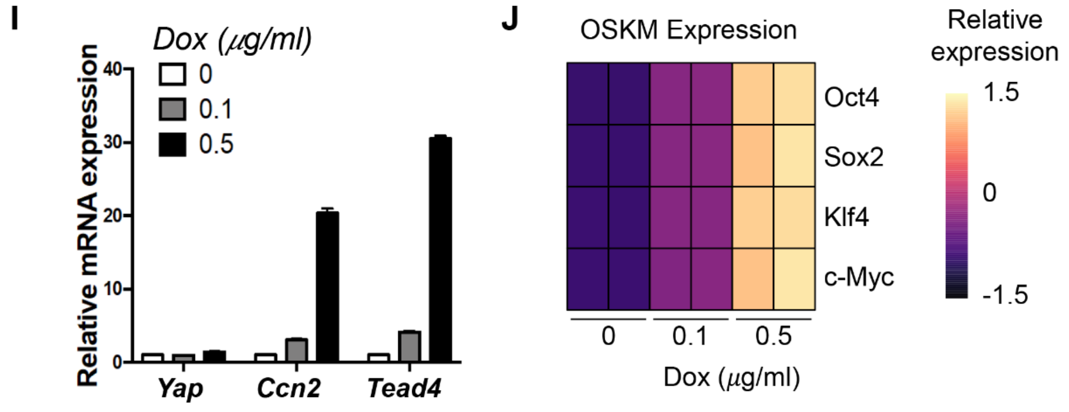

Fig. S2. Concurrent fetal gene transition and YAP activation by partial reprogramming in intestinal organoids (**A**) IF of Oct4 and 2A peptide in iOSKM intestinal organoids. DAPI for nuclear staining. (**B**) Relative mRNA expressions of OSKM in intestinal organoids. (**C**) Relative mRNA expressions of *Nanog*, pluripotency marker, in intestinal organoids. (**D**) Microscopic images of Dox-treated intestinal organoids (left) and quantification of spheroid/budding organoid ratio (right) (n =4). (**E**) Microscopic images of intestinal organoids with Dox treatment for 2days, with Dox treatment for 7 days, and with Dox treatment for 2 days followed by Dox removal for 5 days. (**F**) Microscopic images and IF of fetal genes, Trop2 and Sca1, in intestinal organoids from maternal and E17.5 fetal mice. (**G**) Flow cytometry of Sca1 in Dox-treated intestinal organoids (left) and the percentage (%) of Sca1 positive cells (right). (**H**) Relative mRNA expressions of fetal genes, *Ly6a* (encoding Sca1), *Anxa1*, and *Tacstd2* (encoding Trop2), in iOSKM intestinal organoids. (**I**) Relative mRNA expressions of Hippo pathway-associated genes, *YAP*, *Ccn2* (encoding Ctgf), and *Tead4*, in intestinal organoids. (**J**) Heatmap showing relative expression of OSKM in intestinal organoids.

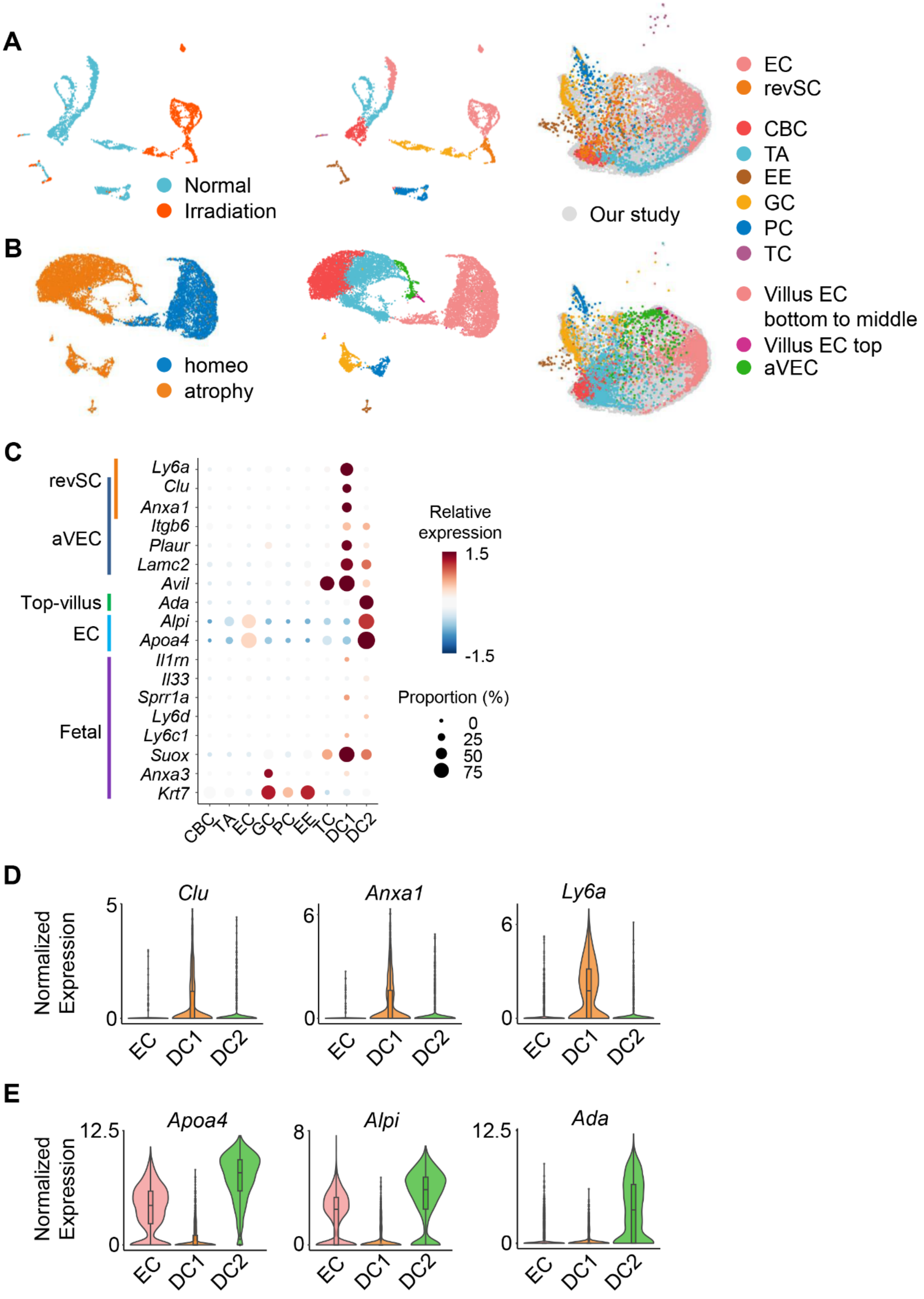

A
Normal
Irradiation
Our study
EC
revSC
CBC
TA
EE
GC
PC
TC
B
homeo
atrophy
Villus EC
bottom to middle
Villus EC top
aVEC
C
revSC
aVEC
Top-villus
EC
Fetal
Ly6a
Clu
Anxa1
Itgb6
Plaur
Lamc2
Avil
Ada
Alpi
Apoa4
Il1rn
Il33
Sprr1a
Ly6d
Ly6c1
Suox
Anxa3
Krt7
CBC
TA
EC
GC
PC
EE
TC
DC1
DC2
Relative
expression
1.5
-1.5
Proportion (%)
0
25
50
75
D
Normalized
Expression
Clu
Anxa1
Ly6a
EC
DC1
DC2
E
Apoa4
Alpi
Ada
12.5
8

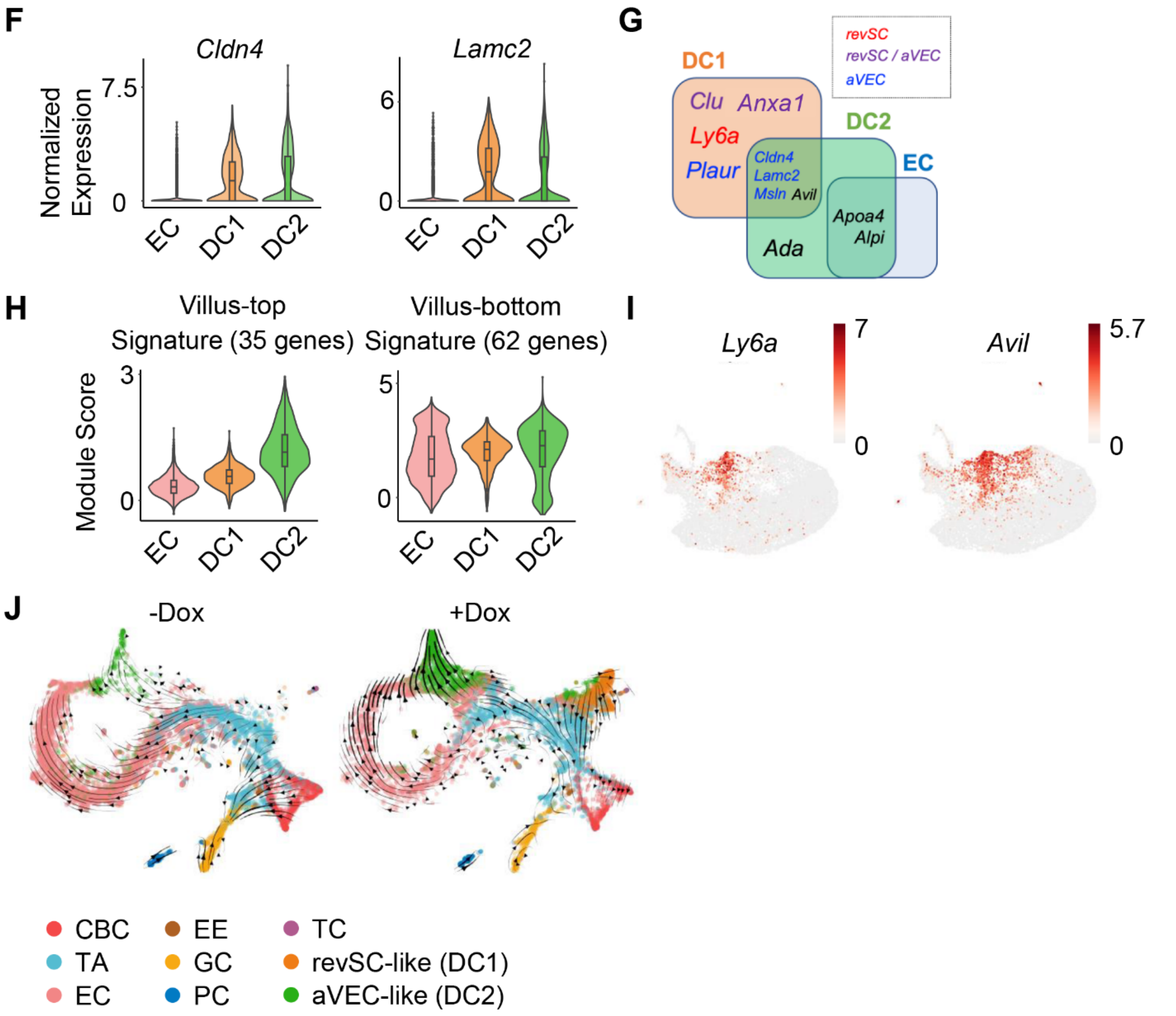

Fig. S3. Characterization of OSKM-induced revSC-like and aVEC-like cells (**A**) UMAP plots showing conditions (left) and cell types (middle) of revSC-containing scRNA-seq data and projection results of revSC-containing scRNA-seq data onto our scRNA-seq data (right). (**B**) UMAP plots showing conditions (left) and cell types (middle) of aVEC-containing scRNA-seq data and projection results of aVEC-containing scRNA-seq data onto our scRNA-seq data (right). (**C**) Relative expression of revSC, aVEC, top villus, EC and fetal gene markers in each cell type, (**D-F**) Violin plots showing expression for revSC (**D**), aVEC (**E**) marker genes, and DC2-specific expressed genes (**F**). (**G**) Venn diagrams showing overlapping cell type-specific marker genes between DC1, DC2 and EC. Colors indicating reported revSC-specific (red), revSC/aVEC common (purple) and aVEC-specific (blue) marker genes. (**H**) Violin plots showing expression for villus-top (left) and villus-bottom (right) EC marker genes. (**I**) UMAP plots showing gene

expression of *Ly6a* (encoding Sca1) and *Avil*. (J) t-SNE plots showing RNA velocity inferred by scVelo in –Dox and +Dox conditions.

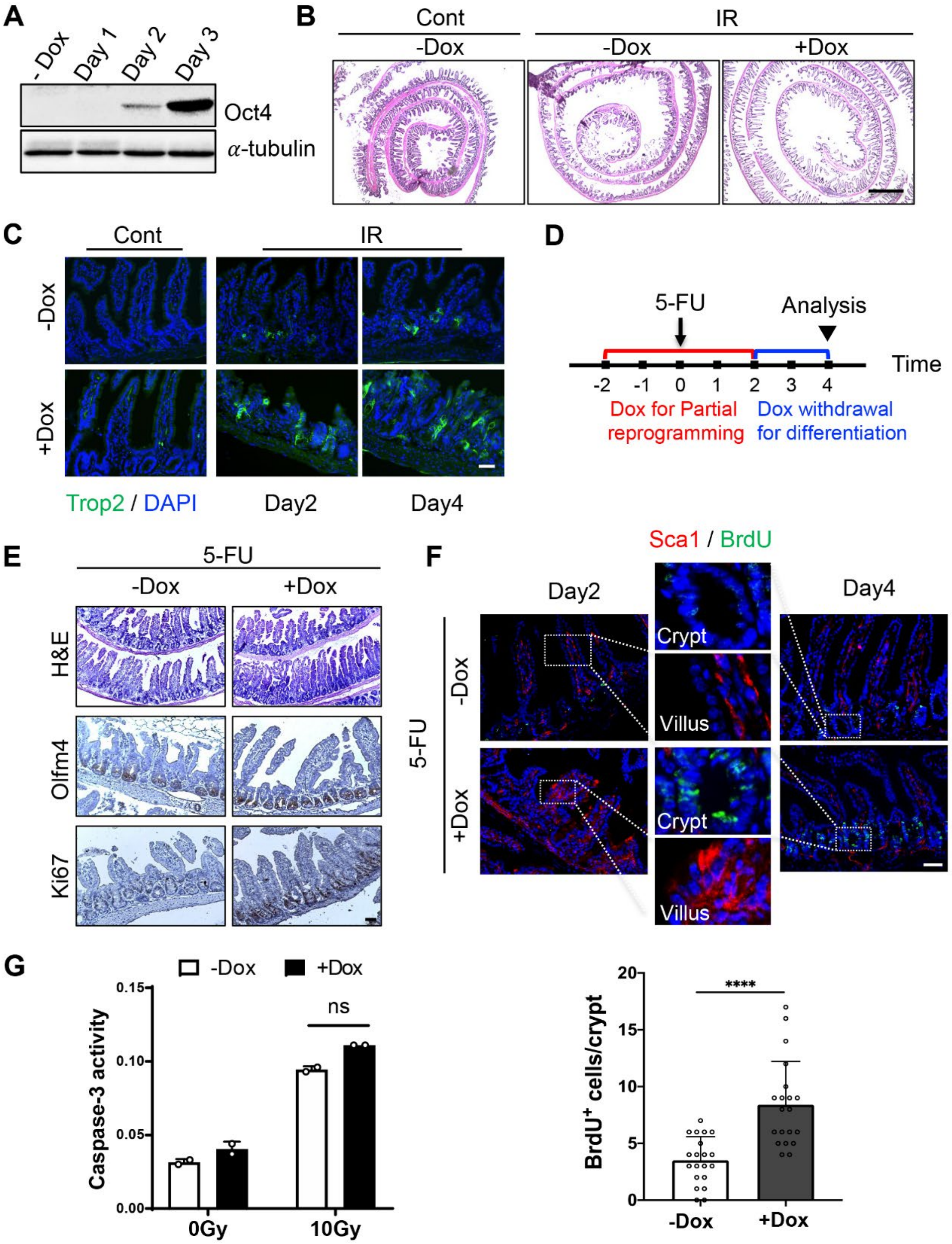

A
- Dox
Day 1
Day 2
Day 3
Oct4
α-tubulin
B
Cont
IR
-Dox
-Dox
+Dox
C
Cont
IR
-Dox
+Dox
Trop2 / DAPI
Day2
Day4
D
5-FU
Analysis
Time
-2
-1
0
1
2
3
4
Dox for Partial reprogramming
Dox withdrawal for differentiation
E
5-FU
-Dox
+Dox
H&E
Olfm4
Ki67
F
Sca1 / BrdU
Day2
Day4
Crypt
Villus
5-FU
-Dox
+Dox
G
-Dox
+Dox
Caspase-3 activity
0.15
0.10
0.05
0.00
ns
0Gy
10Gy
BrdU+ cells/crypt
20
15
10
5
0
****
-Dox
+Dox

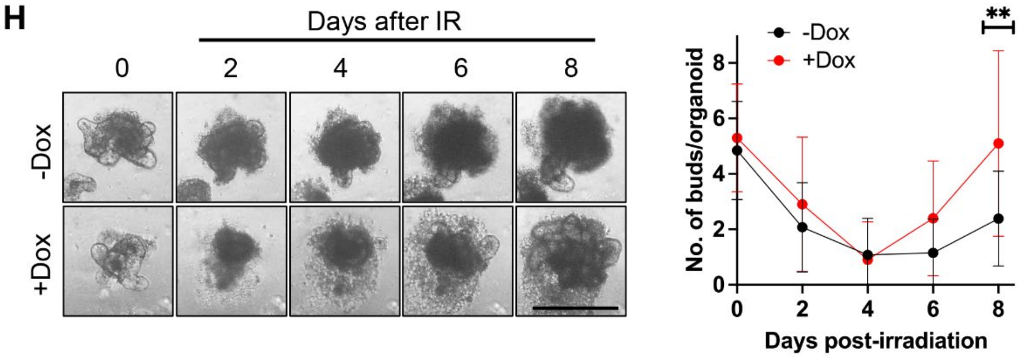

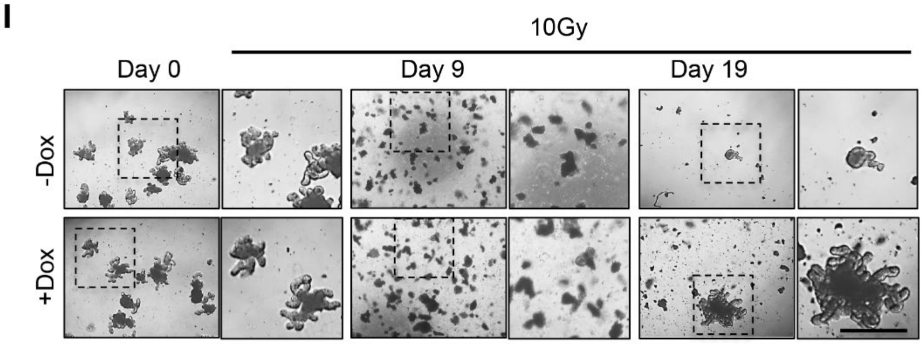

Fig. S4. Promoted intestinal regeneration after IR damage by partial reprogramming (**A**) Western blotting for Oct4 in intestinal epithelial cells of iOSKM mice on Dox treatment for indicated day(s). α-tubulin for loading control. (**B, C**) Intestine of iOSKM mice after 10Gy IR. Dox was treated 2 days before IR for 4 days. H&E histology at 4dpi (**B**) and IF of Trop2 at 2dpi and 4dpi (**C**). (**D**) Experimental scheme for 5-FU and Dox treatment in iOSKM mice. (**E**) H&E histology and IHC for Olfm4 and Ki67 in the intestine of iOSKM 4 days after 5-FU treatment. (**F**) IF of Sca1 and BrdU in the intestine of iOSKM mice 2 days and 4 days after 5-FU treatment (top) and the quantification of BrdU+ cells per crypt (bottom). (n = 2 mice) (**G**) Caspase-3 activity using intestinal organoids 24 hours after IR (n=2) (**H**) Microscopic images of control and Dox-treated intestinal organoids until 8 dpi without passaging (left) and the quantification of the number of buds per organoid after IR (right) (**I**) Microscopic images of intestinal organoids with Dox and IR treatment. The morphology was analyzed on day 9 (2 days after first passage) and day 19 (5 days after second passage) after IR. Data represent the mean with SD. Student's t-test: $p < 0.0001$(****), ns, not significant. Scale bar = 50μm (C, E, and F), 500μm (H and I).

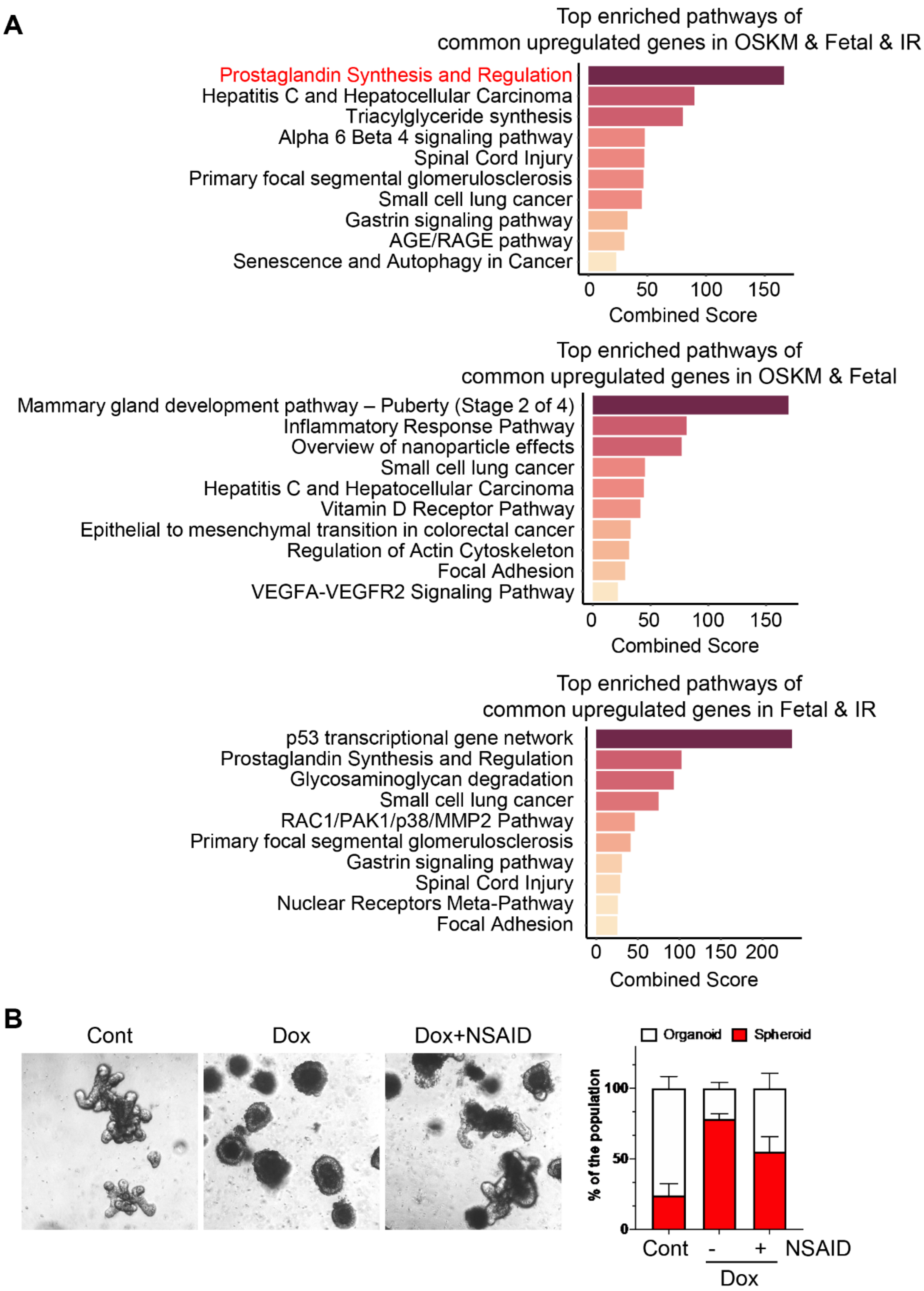
A
Top enriched pathways of
common upregulated genes in OSKM & Fetal & IR
Prostaglandin Synthesis and Regulation
Hepatitis C and Hepatocellular Carcinoma
Triacylglyceride synthesis
Alpha 6 Beta 4 signaling pathway
Spinal Cord Injury
Primary focal segmental glomerulosclerosis
Small cell lung cancer
Gastrin signaling pathway
AGE/RAGE pathway
Senescence and Autophagy in Cancer
0 50 100 150
Combined Score
Top enriched pathways of
common upregulated genes in OSKM & Fetal
Mammary gland development pathway – Puberty (Stage 2 of 4)
Inflammatory Response Pathway
Overview of nanoparticle effects
Small cell lung cancer
Hepatitis C and Hepatocellular Carcinoma
Vitamin D Receptor Pathway
Epithelial to mesenchymal transition in colorectal cancer
Regulation of Actin Cytoskeleton
Focal Adhesion
VEGFA-VEGFR2 Signaling Pathway
0 50 100 150
Combined Score
Top enriched pathways of
common upregulated genes in Fetal & IR
p53 transcriptional gene network
Prostaglandin Synthesis and Regulation
Glycosaminoglycan degradation
Small cell lung cancer
RAC1/PAK1/p38/MMP2 Pathway
Primary focal segmental glomerulosclerosis
Gastrin signaling pathway
Spinal Cord Injury
Nuclear Receptors Meta-Pathway
Focal Adhesion
0 50 100 150 200
Combined Score
B
Cont
Dox
Dox+NSAID
Organoid
Spheroid
% of the population
100
50
0
Cont
-
+
NSAID
Dox

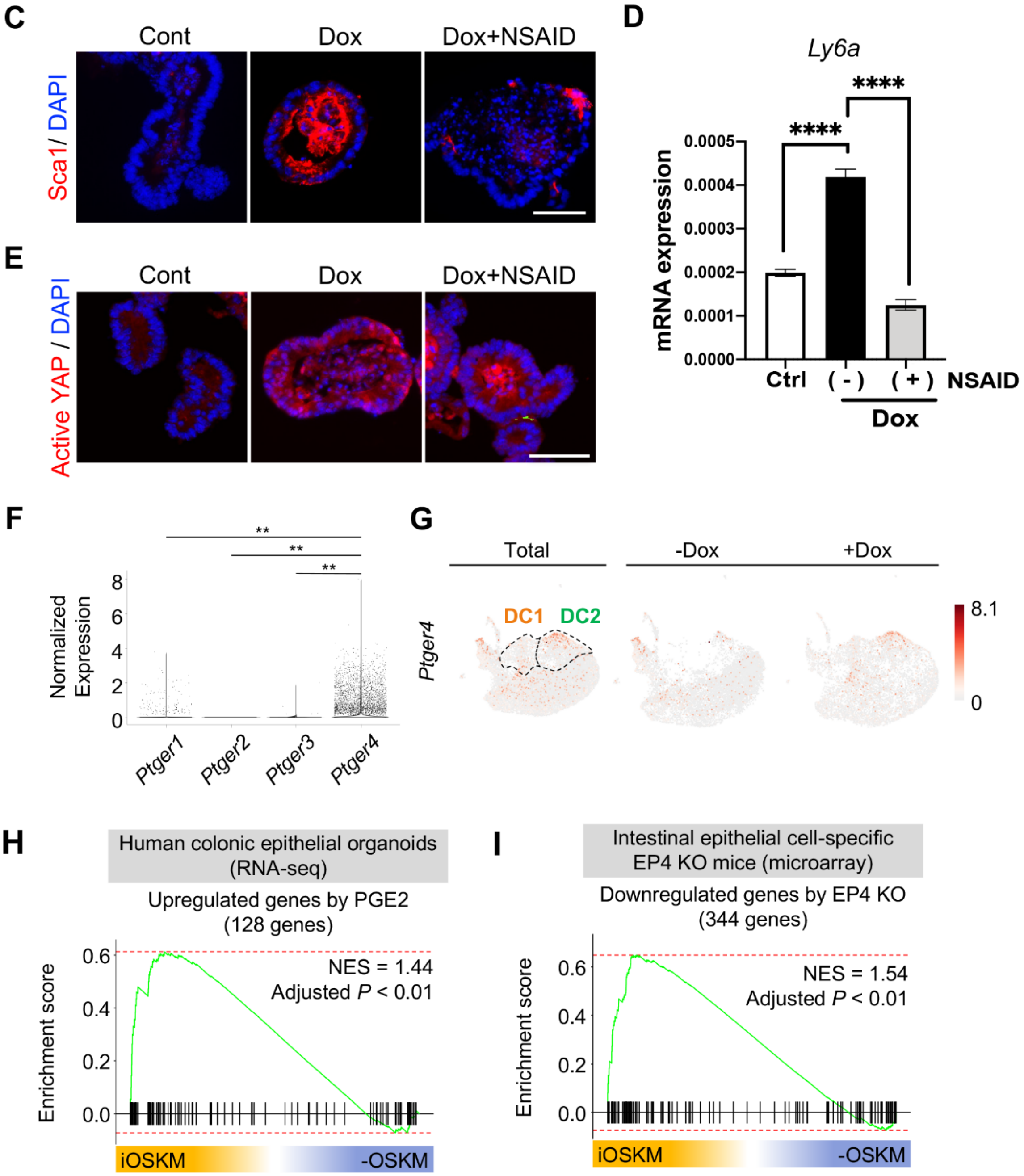

Fig. S5. Inhibition of fetal gene transition and YAP activation by NSAID in intestinal organoids (**A**) Bar plots showing enrichR combined scores of top enriched pathways of common upregulated genes in indicated condition. (**B**) Microscopic images of Dox-treated intestinal organoids with or without NSAID (left) and quantification of budding organoid and spheroid ratio (right) (n =4). (**C**) IF of Sca1 in mouse intestinal organoids. (**D**) mRNA expressions of fetal gene *Ly6a* (encoding Sca1) iOSKM intestinal organoids. (**E**) IF of active YAP in mouse intestinal

organoids. DAPI for nuclear staining. (**F**) Violin plots showing expression for *Ptger1-4* encoding EP1-4 receptors in mouse small intestinal epithelium. (**G**) UMAP plots showing *Ptger4* expression in iOSKM mice (**H, I**) Gene set enrichment analysis (GSEA) results in iOSKM intestinal organoids of up-regulated genes by PGE2 treatment in human colonic epithelial organoids (**H**) and down-regulated genes in intestinal epithelial cell-specific EP4 knockout mice (**I**). Data represent the mean with SD. Student's t-test: $p < 0.01$(**), $p < 0.0001$(****).

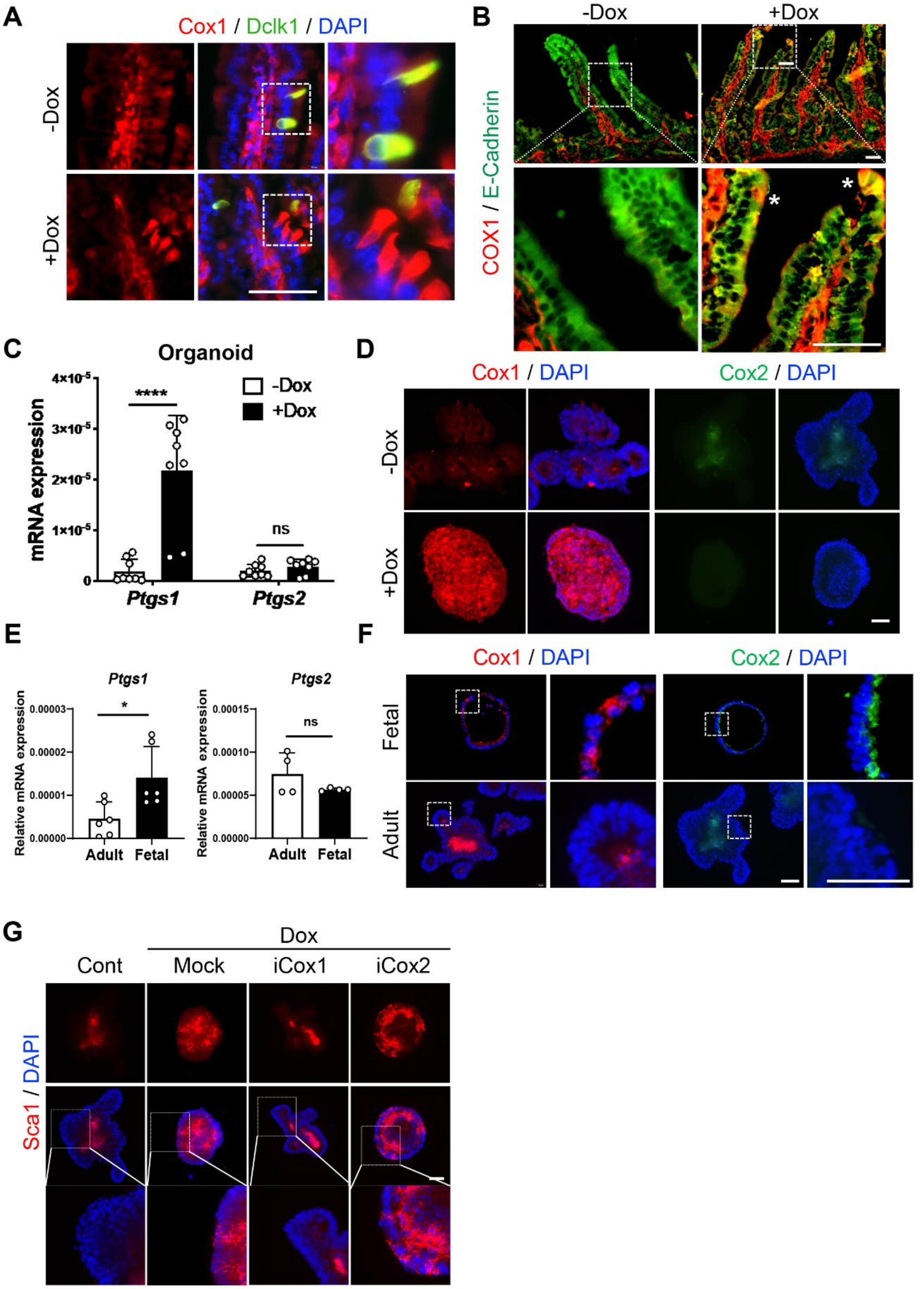

Fig. S6. Increase in Cox1 expression and prime role in intestinal regeneration (**A**) IF of Cox1

and Dclk1(a marker of tuft cells) in the intestine of iOSKM mice. (**B**) IF of Cox1 and E-Cadherin in the intestine of iOSKM mice (**C**) mRNA expressions of *Ptgs1* and *Ptgs2* (encoding Cox1, Cox2 respectively) in iOSKM intestinal organoids (n = 8; 4 x 2 technical replicates). (**D**) IF of Cox1 and Cox2 in iOSKM intestinal organoids. DAPI for nuclear staining. (**E**) mRNA expressions of *Ptgs1* and *Ptgs2* in adult and fetal intestinal organoids (**F**) IF of Cox1 and Cox2 in adult and fetal intestinal organoids. DAPI for nuclear staining. (**G**) IF of Sca1 in iOSKM intestinal organoids. Doxycycline was treated with Cox1 inhibitor (iCox1: SC-560) or Cox2 inhibitor (iCox2: Celecoxib).

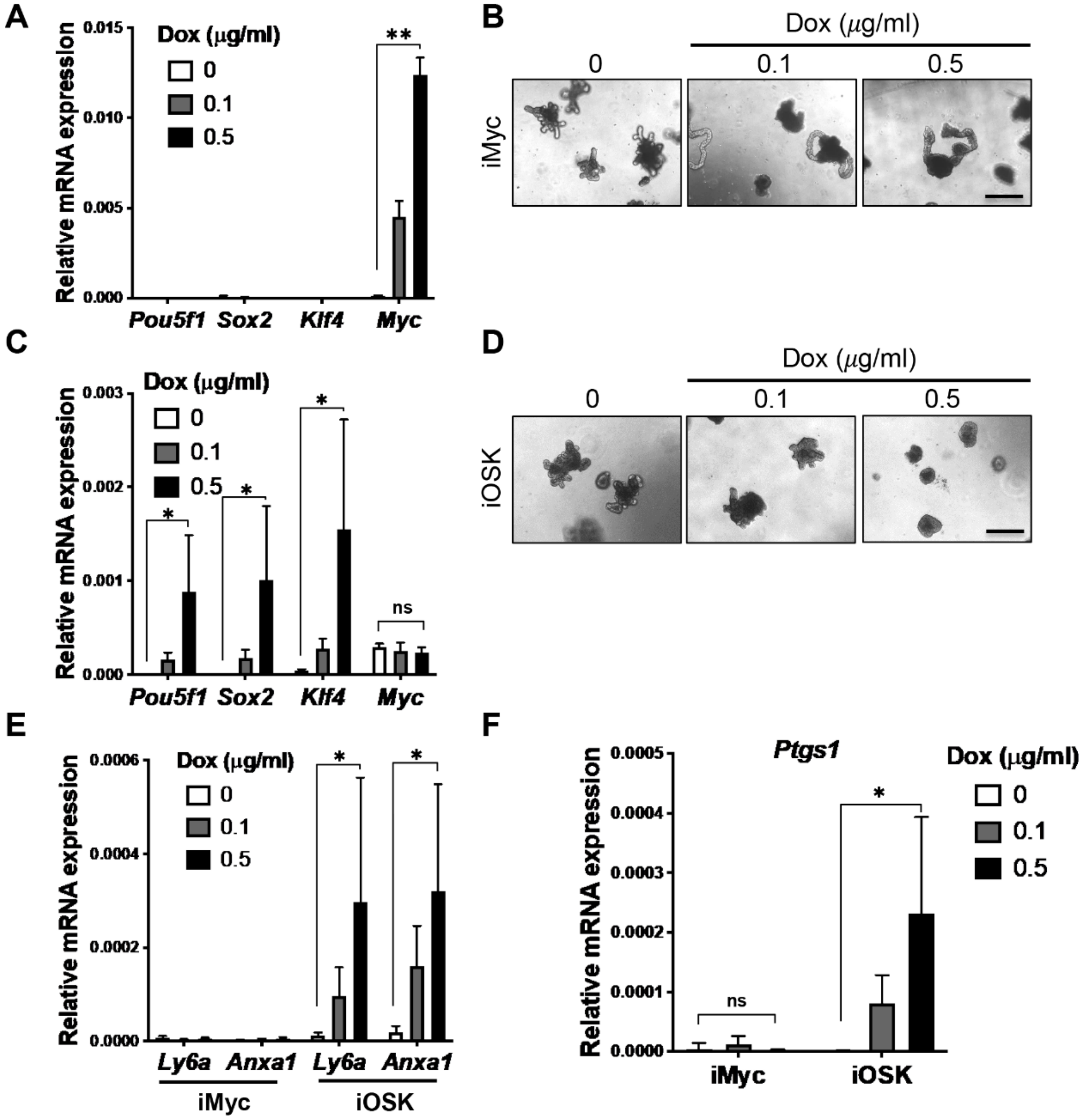

Fig. S7. Lack of Myc in partial reprogramming for intestinal organoids

(**A**) Relative mRNA expression of OSKM in Dox-inducible Myc (iMyc) intestinal organoids (n = 4; 2 x 2 technical replicates). (**B**) Microscopic images of Dox-treated iMyc intestinal organoids (**C**) Relative mRNA expression of OSKM in Dox-inducible OSK (iOSK) intestinal organoids (n = 4; 2 x 2 technical replicates). (**D**) Microscopic images of Dox-treated iOSK intestinal organoids. (**E**) Relative mRNA expressions of fetal genes, *Ly6a* and *Anxa1*, in iMyc and iOSK intestinal organoids (n = 4; 2 x 2 technical replicates). (**F**) mRNA expression of *Ptgs1* in iMyc and iOSK intestinal organoids (n = 4; 2 x 2 technical replicates). Data represent the mean with SD. Student's t-test: $p < 0.05$(*), $p < 0.01$(**), ns, not significant. Scale bar = 50μm (A, F for IF), 500μm (D, E, F for DIC, L and N).

Table S1. Gene signatures used in this study

Movie S1. Growth of intestinal organoids in both conditions, control (A) and Dox treatment (B), for 3 days
